# Interactive room design as a tool for understanding form and style preferences

**DOI:** 10.1038/s41598-025-23543-5

**Published:** 2025-10-13

**Authors:** Kira Pohlmann, Noah Lichtlein, Fariba Mostajeran, Nour Tawil, Simone Kühn

**Affiliations:** 1 Center for Environmental Neuroscience, Max Planck Institute for Human Development, Lentzeallee 94, 14195 Berlin, Germany; 2 Department of Psychology, Humboldt-Universität zu Berlin, Unter den Linden 6, 10099 Berlin, Germany; 3 Department of Informatics, Human-Computer Interaction Group, Universität Hamburg, Vogt-Kölln-Straße 30, 22527 Hamburg, Germany; 4 Clinic and Policlinic for Psychiatry and Psychotherapy, University Medical Center Hamburg-Eppendorf, Martinistr. 52, 20251 Hamburg, Germany; 5 Max Planck UCL Centre for Computational Psychiatry and Ageing Research Berlin, Germany and London, UK, Lentzeallee 94, 14195 Berlin, Germany

**Keywords:** Architectural psychology, Interior design, Living rooms, Forms, Style, Virtual environment, Psychology, Psychology, Science, technology and society

## Abstract

Given the substantial time people spend indoors, it is crucial to understand how aesthetic decisions in interior design are made, both to inform psychological research and guide practical applications. Recent studies show growing interest in form preferences, often suggesting a general preference for curved over angular shapes. Unlike previous research using predefined stimuli, our study allowed individuals to freely express their preferences in a living room through an interactive 3d web application. In an online experiment, participants (*N* = 196) furnished a virtual living room according to their personal preferences, using a controlled set of furniture items that varied systematically in form (angular vs. curved) and style (modern vs. classic). Contrary to prior findings, we observed a small but significant overall preference for angular furniture. This effect was mainly driven by male participants, who selected more angular than curved items, while female participants showed no form preference. We found no general preference in terms of style. Additionally, we explored associations between design preferences and personality traits (BFI-2), but no significant relationships emerged. Overall, this study demonstrates the value of interactive 3d environments for investigating aesthetic preferences in a controlled yet flexible setting, allowing personalised design decisions beyond predefined conditions.

**Supplementary Information:**

The online version contains supplementary material available at 10.1038/s41598-025-23543-5.

## Introduction

A substantial portion of daily life is spent indoors. In European countries, people typically spend only one to two hours outdoors per day, meaning the majority of their time is spent inside enclosed spaces^1^. Previous studies found that individuals living in Canada, the United States, and Germany spend approximately 65% of their time indoors at home^2,3^. Given this extensive indoor exposure, the characteristics of built environments, particularly the living space, are suggested to play a crucial role in shaping human well-being and behaviour^4^.

Various design elements influence how indoor spaces are experienced, including factors such as lighting, colour, and spatial geometry^4^. For instance, colour can impact cognitive processes and task performance^5^. Additionally, the interior design style contributes to the perception and experience of spaces and furniture^6–8^.

One key design feature that influences aesthetic perception and emotional responses is architectural form. Research has consistently shown a preference for curved over angular shapes in abstract forms, with curvature being perceived as visually pleasant^9,10^. However, the effect of curvature is not uniform across all stimuli—while curved shapes are generally favoured in stimuli with neutral or positive valence, this preference diminishes when stimuli convey negative emotions^11^. Beyond abstract shapes, curvature also plays a role in real-world design evaluations. In car design, for instance, curved features have been shown to enhance perceptions of attractiveness^12^. Similarly, studies explicitly measuring liking have found that people consistently rate curved objects more positively than angular ones^13^.

The preference for curvature extends beyond abstract shapes and everyday objects to architectural spaces and furniture design^8,14,15^. Studies suggest that curved furniture and interior elements are often associated with more positive aesthetic evaluations. For example, in a study using grayscale computer-generated images of living room scenes, participants rated rooms with curved furniture as more pleasurable than those with angular furniture^14^. Similarly, a study using functional magnetic resonance imaging (fMRI) found that interior spaces featuring curvilinear design elements were perceived as more beautiful, with greater activation in brain regions associated with reward processing when participants viewed such environments^15^.

In contrast, Bar and Neta^16^ found that images of sharp-angled objects elicited stronger amygdala activity – a brain region associated with fear processing – compared to curved objects, suggesting that sharp edges might be perceived as potential threats on an implicit or explicit level. Their study included everyday objects, such as sofas and plants, highlighting that this effect may generalise beyond abstract forms. However, not all studies consistently support a preference for curvature. In a virtual reality (VR) experiment where participants freely explored living rooms with either curved or angular furniture, no clear preference for curvature emerged^7^. Interestingly, when images of these same living rooms were evaluated in an online experiment, participants rated spaces with curved furniture higher in terms of beauty and liking than those with angular furniture^8^. These findings suggest that while curvature is often preferred, contextual factors—such as the mode of presentation and level of immersion—may influence how it is perceived.

Beyond form, style also represents a relevant feature in the context of architecture^6,17,18^, with a common distinction made between a modern, more simplistic style and a classic, more traditional style. For instance, Ozkan and Yildirim^18^ found that contemporary-style hotel guestrooms were evaluated more positively than traditional-style rooms. In contrast, Mastandrea and Maricchiolo^6^ reported no preference for modern over classic chairs among laypeople, whereas design experts favoured modern chairs. Similarly, a VR study on living rooms found no differences between modern and classic styles in participants’ judgments of pleasantness and beauty^7^. To increase the generalisability of findings on form preferences, some studies have incorporated style as a secondary variable^7,14^.

While many studies have identified a general preference for curvature, interindividual differences, such as expertise in art or architecture, or sex, also play a role in shaping aesthetic judgments. For instance, architectural expertise appears to influence contour preferences. When evaluating the beauty of images of architectural spaces, self-identified experts in architecture and design showed a clear preference for curvilinear spaces, whereas effects diminished in non-experts^19^. However, when asked whether they would like to enter these spaces, the pattern reversed and non-experts preferred to enter curvilinear spaces, while experts showed no significant preference.

Gender differences have also been observed in studies on contour preferences, e.g., Palumbo et al.^20^ found that female psychology students exhibited the strongest preference for curved shapes. Similarly, in a VR study, male participants demonstrated better cognitive performance in rooms with angular furniture compared to female participants^7^. They also rated angular conditions more positively than female participants. This suggests that forms may influence affect and cognition differently across genders. Further supporting this notion, an Implicit Association Task revealed that female participants showed a stronger tendency to approach curved designs and avoid angular ones, whereas this effect was less pronounced in male participants^21^. Additionally, when rating images of living rooms, female participants expressed higher beauty and liking ratings for curvilinear interiors, while no significant preferences were observed among male participants^8^. These findings highlight that while curvature is often favoured, individual differences, particularly in expertise and sex, can modulate this preference.

In addition, personality plays a significant role in shaping preferences for indoor design, as individuals often express aspects of their identity through their living spaces^22^. One widely used framework for measuring personality traits is the Big Five Inventory^23^, which was later refined into the Big Five Inventory 2 (BFI-2)^24^. The BFI-2 assesses personality along five dimensions: extraversion (e.g., outgoing, active, and social), agreeableness (e.g., compassionate, respectful, and trustworthy), conscientiousness (e.g., organised, productive, and responsible), negative emotionality (previously termed neuroticism, referring to tendencies toward stress and emotional instability), and open-mindedness (formerly openness, encompassing intellectual curiosity, aesthetic sensitivity, and creative thinking).

Several studies have explored how personality traits influence aesthetic preferences in interior spaces. For instance, a VR study investigating the effect of linear and curved geometries on pleasure and arousal ratings found that personality played a moderating role. Among the reported effects, participants scoring higher in neuroticism (negative emotionality) tended to rate curved environments as less pleasurable, whereas those with high agreeableness showed increased pleasure ratings for curvature^25^. Beyond spatial geometry, personality traits have also been linked to material preferences, with certain traits predicting consumer preferences for wooden furniture^26^. Similarly, when apartment interiors were designed to reflect certain personality traits and presented to participants in VR, the perceptions of the imaginary apartment tenants aligned with the personality traits that guided the design process of the interiors^27^. Further, judgments on interior spaces were linked to specific cues in the presented environments^28^. In their study, Gosling et al.^28^ demonstrated that observers formed consistent personality judgments based on offices and bedrooms, using cues such as organisation and decoration as the basis for their impressions. Additionally, personality traits have been shown to predict preferences for interior spaces; for example, introverts tended to favour enclosed spaces with high-contrast colour schemes^29^. These findings suggest that personality not only influences how individuals experience architectural and interior spaces but also how they decide to furnish their own living environments and project their identities onto their surroundings.

Importantly, most studies investigating contour preferences in interior design have exposed participants to predefined conditions, often presenting exaggerated contrasts between entirely angular or entirely curved environments^7,14^. However, such extremes are rarely encountered in real-life settings, where individuals typically incorporate a mixture of both design elements. To address this limitation, we propose a novel approach that allows participants to actively shape their environment rather than passively evaluate predefined stimuli. In our study, participants were provided with a limited selection of furniture items and tasked with furnishing an empty living room according to their personal preferences. This was implemented as a 3d web application, enabling users to navigate the space from both a first-person and bird’s-eye perspective, similar to visualisations commonly seen in video games.

This interactive approach not only provides insight into general form preferences but also allows for a more nuanced analysis of category-specific choices, e.g., whether preferences for curvature varied across different furniture types, such as sofas versus armchairs. In this study, we took the rate of choosing one condition over the other as a proxy for preference. Additionally, by linking participants’ furnishing choices to their personality traits, we aimed to explore whether individual differences in personality influence interior design preferences. By allowing participants to design living environments themselves, our method offers a more ecologically valid framework for studying the relationship between personality and interior design preferences, specifically focusing on form and style preferences.

## Materials and methods

We developed a 3d interactive web application enabling participants to furnish an empty virtual living room using a predefined set of furniture items. This approach is similar to mobile games that let users design virtual spaces; however, our application provided control over several key aspects. Participants could freely select and arrange furniture, with no restrictions on the number of items they could use. At the same time, we ensured experimental control by providing a balanced and systematically structured set of items, allowing us to investigate individual design preferences under controlled conditions. The entire experiment was conducted in the German language. Therefore, all materials, including the questionnaires and the texts in the developed application, were presented in German.

### Conditions

We provided participants with a balanced set of furniture items, allowing them to furnish their ideal living room freely. The furniture set was systematically controlled along two key dimensions: form (angular vs. curved) and style (modern vs. classic).

- Form: Angular items predominantly featured 90° angles, while curved items had no hard edges.
- Style: Modern items followed a minimalistic design, whereas classic items were more detailed, including embellishments and ornamentations.

Each furniture item was characterised by a combination of form and style, resulting in four distinct categories: angular-modern, angular-classic, curved-modern, and curved-classic. To ensure a controlled comparison, we designed each item to have a matching counterpart within its category. For instance, the angular-modern armchair had a corresponding curved-modern version that was identical in all aspects except for its form. Additionally, all items within each category were size-matched to maintain consistency across conditions. We used the same 3d furniture objects that were employed in previous studies to investigate form preferences in interior design^7,8,21^. We added two additional sets of furniture, matching the previous ones, to offer participants a larger selection.

### Hypotheses

If participants consistently chose one form or style over the other, we interpreted this as an indication of a preference. In the absence of such a preference, we expected their choices to be distributed evenly across categories (e.g., 50% angular, 50% curved). We hypothesised that participants would prefer curved over angular objects, selecting a higher proportion of curved items. Building on previous research suggesting sex-related differences in contour preferences^8,20^, we further hypothesised that female participants would show a stronger preference for curved furniture than male participants.

We treated style (modern vs. classic) as an exploratory factor without a directed hypothesis, given inconsistent findings in prior research^6,8,18^. We also examined in an exploratory manner whether preferences for form and style varied across different furniture categories. Finally, we hypothesised that personality traits would be associated with participants’ form and style preferences.

### Interactive web application

**Fig. 1:**
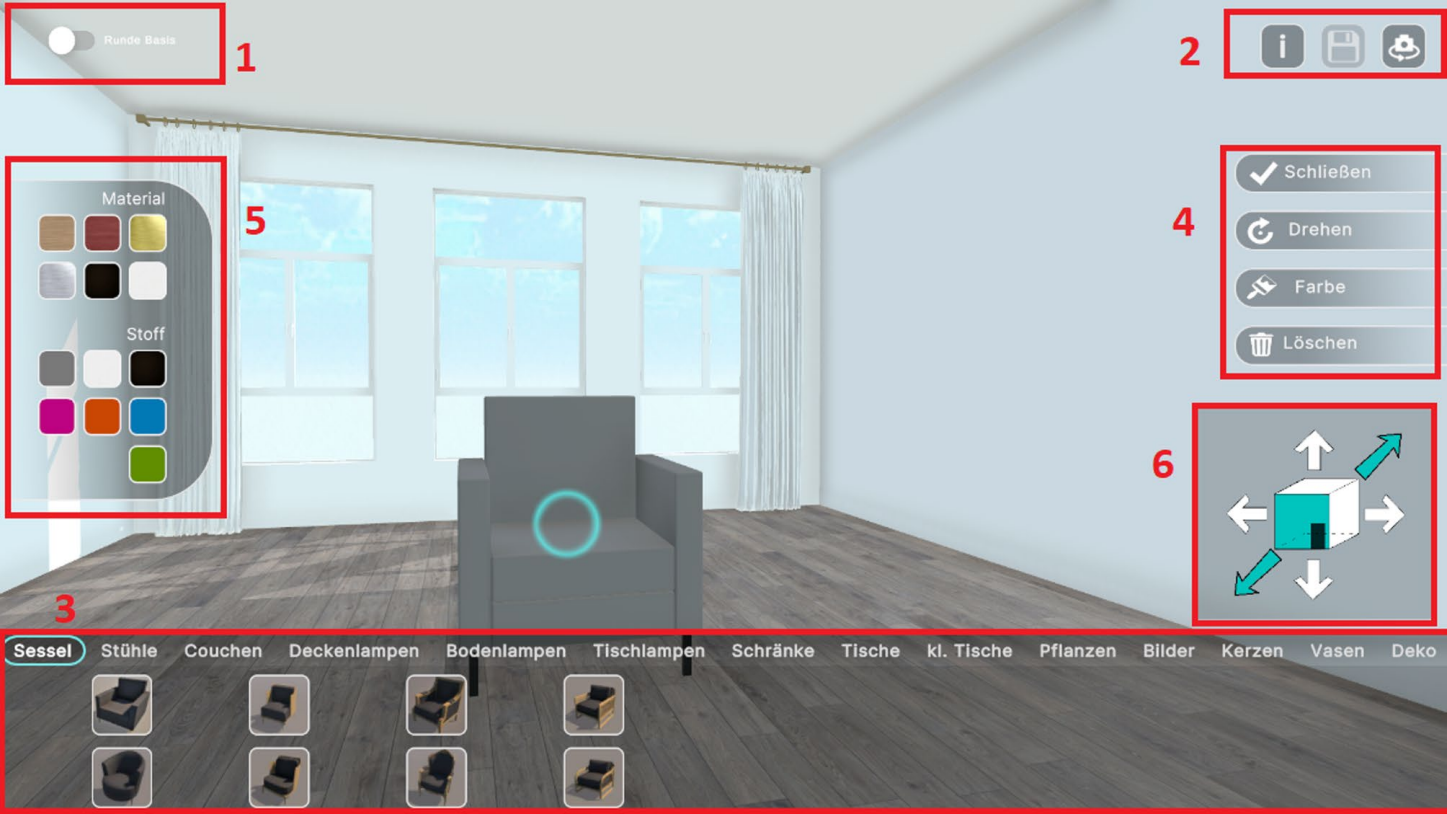
User Interface of the 3d web application. 1: Toggle switch to change the form of the windows; 2: information button for instructions; save button (accessible from 30 min onwards); camera button to switch between bird’s eye and first person view; 3: furniture categories and items to select from; from left to right: armchairs, chairs, sofas, ceiling lamps, floor lamps, table lamps, cabinets, tables, small tables, plants, paintings, candles, vases, and decorations; 4: action buttons to confirm items status, rotate, change colour or delete item; 5: options to modify material and (if applicable) fabric of the item; 6: buttons to move the selected item within the room.

The interactive web application was developed using Unity (version 2022.2.3f1, https://unity.com). The application centred around an empty virtual living room that participants could furnish freely. An overview of the web application is provided in Fig. 1, highlighting all action panels. The room had a size comparable to 7.4 × 5.4 m, and a height of 3 m, featuring a door in one corner and three windows on the opposite wall. The window view was kept neutral to avoid any bias it might have on the furniture placement within the room. Participants could navigate the space using the keyboard keys W, A, S and D and adjust their camera perspective by holding the right mouse button. A camera button in the top-right corner allowed switching between bird’s eye and first-person views (see Panel 2 of Fig. 1). Additionally, a toggle button in the top-left corner enabled participants to switch between angular and curved versions of the windows and door trims (see Panel 1 of Fig. 1). The option to modify the form of these room elements was included for consistency with preceding studies using similar stimuli^7,8^. A video demonstrating this application can be found here: https://doi.org/10.17605/OSF.IO/27TXP.

#### Furniture selection and categorisation

Furniture items were displayed at the bottom of the screen (see Panel 3 of Fig. 1), organised into the following categories: armchairs, chairs, sofas, ceiling lamps, floor lamps, table lamps, cabinets, tables, small (side) tables, plants, paintings, candles, vases, and decoration (which included baskets, carpets, and cushions). Most categories contained eight items, with two per form-style combination (angular-modern, angular-classic, curved-modern, curved-classic). Exceptions included the table lamps and paintings, which had only one item per form-style combination (four in total). Decorative items (baskets, carpets, and cushions) had only one version per form, without a style distinction, and plants had one angular-shaped and one curvilinear-shaped option, but with varying pot styles and materials. A full list of all available furniture items (102 in total) is provided in the supplementary material.

#### Interaction and customisation

Participants could select furniture items from the interface at the bottom of the screen. Upon selection, the item appeared in the centre of the room, marked by a blue circle indicating it was active for modification. Participants could then move the item freely within the room, rotate the item in 90° increments, and change its material and fabric (if applicable). Once an item was positioned, participants could confirm their placement and close the action buttons (see Panel 4 of Fig. 1). However, previously placed items could be selected and modified again at any time.

#### Material and fabric options

A limited selection of materials and fabrics was offered (see Panel 5 of Fig. 1). For materials, we included two wood options (light and dark wood), two metal options (silver and gold), and two neutral options (black and white). For fabric, we included three neutral options (grey, white, and black), two warm colours (pink and orange), and two cold colours (blue and green). All fabric colours were matched for luminance to ensure equal contrast across conditions. While the material selection was available for all furniture items (besides carpets), the fabric selection was only available for upholstered items (such as sofas and armchairs) and carpets.

### Participants

Ethical approval for this study was obtained from the local ethics committee at the Center for Psychosocial Medicine, University Medical Center Hamburg-Eppendorf, in Hamburg, Germany (reference number: LPEK-0657). The experiment was conducted online and hosted on the Prolific platform (https://www.prolific.com), where it was made available to participants residing in Germany who were of legal age. A G*Power^30^ analysis indicated a sample size of *N* = 147 for a one-sample t-test with an expected effect size of 0.3. Due to anticipated exclusions due to technical errors, we decided to aim for 200 participants after applying exclusion criteria. At the beginning of the experiment, participants were required to confirm that they had no neurological or psychological disorders, were not affected by red-green blindness, and would use visual aids (e.g., glasses) if needed. We excluded participants if they: withdrew consent during the study, placed furniture in an illogical or nonsensical manner (e.g., a sofa facing a wall), or furnished the room with fewer than four items (although there was no strict minimum requirement). For some participants, we experienced technical issues that resulted in missing data on furniture placement. Hence, we excluded these participants from further analysis. A total of 263 participants completed the experiment. After applying exclusion criteria, the data of 67 participants were removed (63 participants due to technical issues resulting in missing data on furniture placements and four participants due to illogical placement of the furniture), leaving 196 participants’ data for further analysis. Of these, 99 indicated male as their sex assigned at birth (M_age_ = 29.6, SD = 8.33), with ages ranging from 18 to 59 years, and 97 indicated female (M_age_ = 29.2, SD = 8.54), with ages ranging from 19 to 63 years. In total, four participants identified differently from their sex assigned at birth. For the subsequent analysis, we focused on sex assigned at birth; therefore, any references to sex or sex-related differences pertain to sex assigned at birth. Participants were compensated with 6 GBP upon completion of the experiment.

### Procedure

At the beginning of the experiment, participants were introduced to the study procedure and data handling policies. After providing informed consent, they completed a tutorial that guided them through the web application’s functionalities. The tutorial explained how to navigate the room, select and place furniture, and adjust materials and fabrics. Participants were instructed to design a living room according to their personal preferences, and they could revisit the tutorial at any time by clicking the information button in the top-right corner of the application.

Once the tutorial was completed, participants proceeded to the furnishing task, on which they were instructed to work for at least 30 min. During this period, they could freely arrange and customise the furniture in the virtual living room. To ensure that all participants had sufficient time to engage with the task, the option to save and proceed was only enabled after the full 30 min had elapsed.

**Fig. 2:**
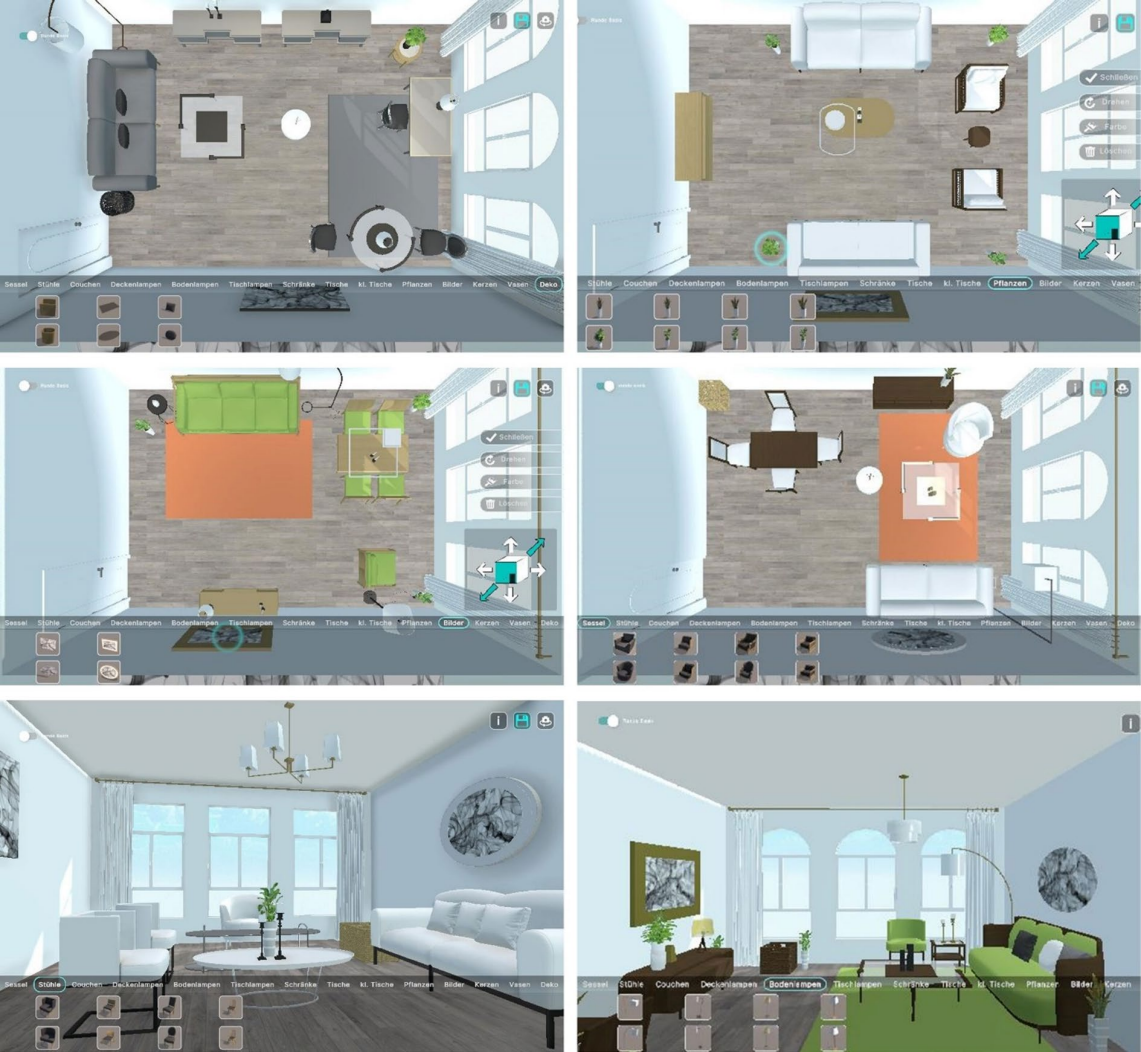
Examples of furnished rooms from study participants. The top and middle rows show rooms from a bird’s-eye view and the bottom row from a first-person view. Participants were free to switch between these perspectives at any time. The screenshots of the final rooms were taken after participants spent at least 30 min furnishing the rooms and clicked on the save button to proceed with the experiment.

After completing the furnishing task, participants were directed to a questionnaire implemented using Inquisit by Millisecond (version 6.6.1, https://www.millisecond.com). The questionnaire collected demographic information, details about participants’ current and preferred living environments, and responses to an adapted version of the Vienna Art Interest and Art Knowledge Questionnaires^31^, modified to focus on architecture^21^. We included the VAIAK to assess individual differences in knowledge in art and architecture, building on previous research showing that form preferences can differ between experts and non-experts^19^. To assess personality traits, we used the German version of the BFI-2^24,32^, which consists of 60 items rated on a five-point Likert scale (1 = strongly disagree; 2 = disagree a little; 3 = neutral; no opinion; 4 = agree a little; 5 = strongly agree). The entire experiment took approximately 40 min to complete. Examples of some resulting furnished rooms are provided in Fig. 2.

### Data analysis

This study was preregistered (https://aspredicted.org/7rs4-jhg7.pdf). To quantify individual preferences, we calculated the percentage of selected curved items and modern items for each participant. Since each furniture item was categorised as either angular or curved, and either modern or classic, these percentages indicate whether participants exhibited a preference for one category over the other. While the application also allowed changing the form of the windows and door frames (see Panel 1 of Fig. 1), these options were not part of the main furnishing interface, and due to a bug in the application, participants’ choices were not saved; therefore, this data was not included in the analyses. To test for overall preferences, we conducted two-sided one-sample t-tests against 0.5 (indicating no preference). Further, we conducted two-sample two-sided t-tests comparing form and style preferences between male and female participants. Additionally, within each subgroup, we performed one-sample t-tests against 0.5 to test for preferences among male and female participants separately.

We summarised the data by furniture type, form, and style and conducted chi-square tests to evaluate whether form and style preferences varied significantly across different furniture types.

Finally, we conducted multiple linear regression analyses, using the percentage of selected curved items as the dependent variable and scores from the five BFI-2 dimensions (extraversion, agreeableness, conscientiousness, negative emotionality, and open-mindedness) as independent variables. We repeated this analysis with the percentage of selected modern items as the dependent variable.

Due to a technical issue during data collection, only 50 of the 60 BFI-2 items were initially recorded. To address this, we contacted participants after the experiment and asked them to complete the missing 10 items, offering a 1 GBP bonus payment for participation. However, only 100 participants provided the missing responses. As a result, analyses involving BFI-2 scores were conducted on this subset of *N* = 100 participants with complete personality data.

All analyses were conducted in R Studio (version 2024.09.1, R version 4.3.0).

## Results

### General preference for form and style and Sex-related differences

We took the percentage value for curved furniture and ran a two-sided one-sample t-test with mu = 0.5, assuming that if there is no preference, participants would select equal amounts of angular and curved furniture. The test was significant (*t*(195) = −2.66, *p* = 0.008, 95% CI [0.437, 0.491], *d* = −0.19, *M*_*curved*_ = 0.464), indicating, however, that angular furniture was selected more often than curved furniture.

**Fig. 3:**
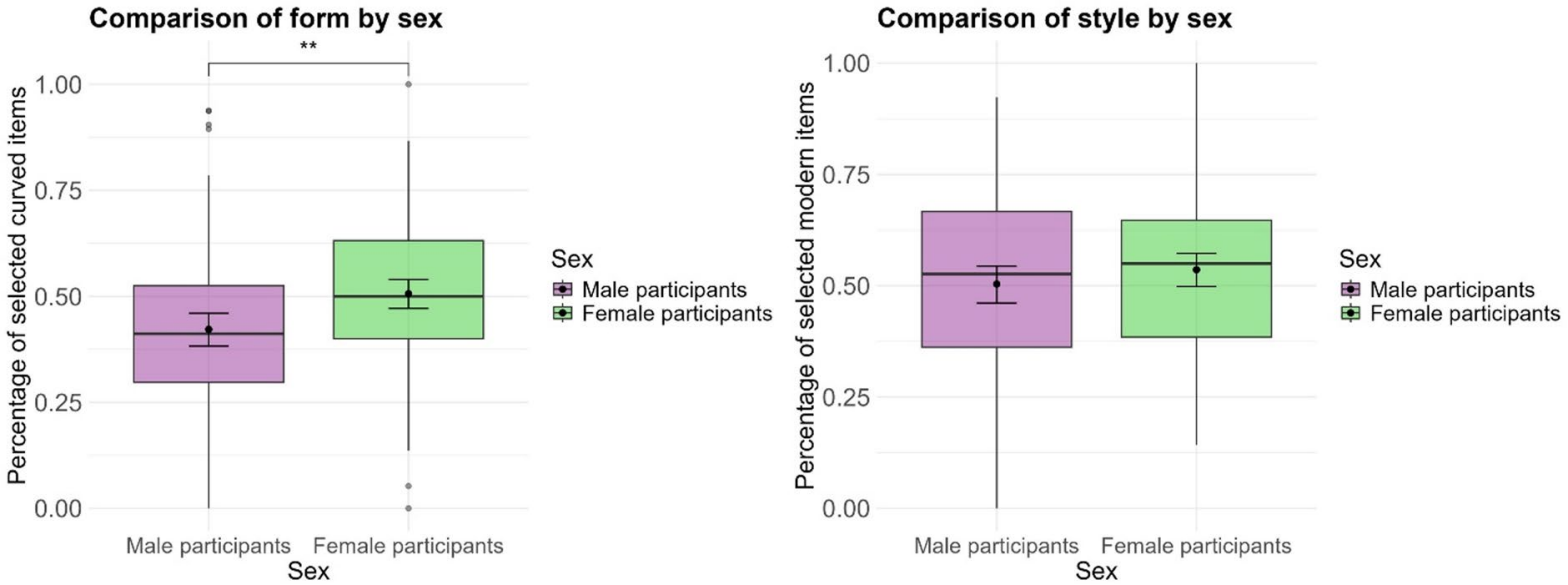
Comparison of the percentage of selected curved items (left) and modern items (right) between male (*N* = 99) and female (*N* = 97) participants. Boxplots display the median and interquartile range, while black dots represent group means. Error bars indicate 95% confidence intervals around the mean. A significant difference was found between the groups (*p* = 0.002) for the percentage of selected curved items. Significant differences are marked with asterisks (*** for *p* < 0.001, ** for *p* < 0.01, and * for *p* < 0.05).

Using the same percentage value representing the percentage of selected curved furniture items, we ran a two-sided t-test between male and female participants. The test was significant (*t(*193.32) = −3.170, *p* = 0.002, *d* = −0.45) and the results are displayed in Fig. 3. On average, male participants had a lower percentage of curved items (*N* = 99, *M*_*curved*_ = 0.422, *SD* = 0.193) than female participants (*N* = 97, *M*_*curved*_ = 0.506, *SD* = 0.178). The 95% confidence interval for the mean difference ranged from − 0.136 to −0.032.

We repeated the two-sided t-test with mu = 0.5 for the male and female sub-samples to test whether there were any preferences for form within the given sub-sample. The test was not significant for the female sub-sample (*N* = 97, *t*(96) = 0.35, *p* = 0.728, 95% CI [0.470, 0.542], *M*_*curved*_ = 0.506). The test was, however, significant for the male sub-sample, showing that they selected more angular items than curved (*N* = 99, *t*(98) = −4.01, *p* < 0.001, 95% CI [0.384, 0.461], *d* = −0.40, *M*_*curved*_ = 0.422,).

We took the percentage value for modern furniture and ran a two-sided one-sample t-test with mu = 0.5, assuming that if there is no preference, participants would select equal amounts of modern and classic furniture. The test was not significant (*t*(195) = 1.360, *p* = 0.175, 95% CI [0.491, 0.548], *M*_*modern*_ = 0.520).

Using the same value representing the percentage of selected modern furniture items, we ran a two-sided t-test between male and female participants. The test was not significant (*t*(190.53) = −0.112, *p* = 0.268, see Fig. 3). On average, male participants did not select fewer or more modern items (*N* = 99, *M*_*modern*_ = 0.504, *SD* = 0.218) than female participants (*N* = 97, *M*_*modern*_ = 0.536, *SD* = 0.186). The 95% confidence interval for the mean difference ranged from − 0.089 to 0.025.

Given the presence of sex-related differences, we looked at age-related differences; however, we did not find any significant effects for age-related differences concerning form or style preferences (full details are provided in the supplementary material). Following Vartanian et al.^19^, who showed that form preferences can differ between experts and non-experts, we calculated a summary score for participants’ VAIAK responses and correlated it with the percentages of selected curved and modern items. A Pearson correlation revealed a small but significant positive association between the percentage of curved items and the VAIAK summary score (*r*(194) = 0.16, *p* = 0.022, 95% CI [0.02, 0.30]). In contrast, no significant association was found between the percentage of modern items and the VAIAK score (*r*(194) = 0.07, *p* = 0.318, 95% CI [−0.07, 0.21]). These results suggest that participants with greater knowledge and interest in art and architecture tended to select slightly more curved items; however, style was unrelated to VAIAK scores.

### Form and style preferences per furniture category

**Fig. 4:**
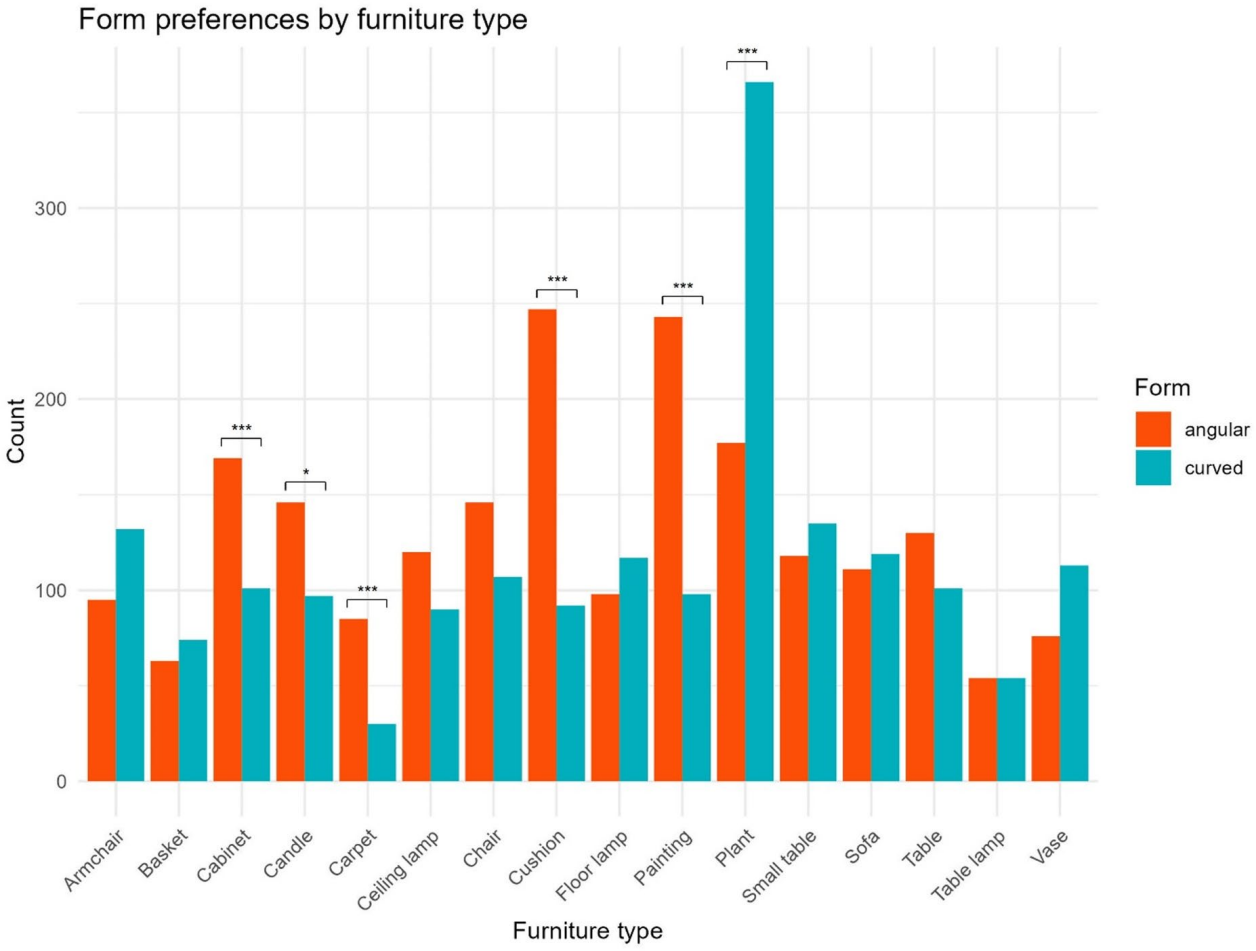
Form preferences by furniture category. Bars indicate the overall count of selected items across all participants for form (angular and curved) and category. Significant differences are based on adjusted p-values and marked with asterisks (*** for *p* < 0.001, ** for *p* < 0.01, and * for *p* < 0.05).

Chi-square tests were conducted to assess form preferences (angular vs. curved) for each furniture type. Significant differences were observed for several furniture types, indicating a preference for one form type over another. The p-values were adjusted using the Bonferroni correction to control for multiple comparisons. Complete results for the chi-square test can be found in the supplementary material. Based on adjusted p-values, the test indicated a preference for angularity for cabinets (*p* < 0.001), candles (*p* = 0.027), carpets (*p* < 0.001), paintings (*p* < 0.001), and cushions (*p* < 0.001), while it indicated a preference for curvature for plants (*p* < 0.001). Figure 4 shows the count of selected angular and curved items by furniture category.

**Fig. 5:**
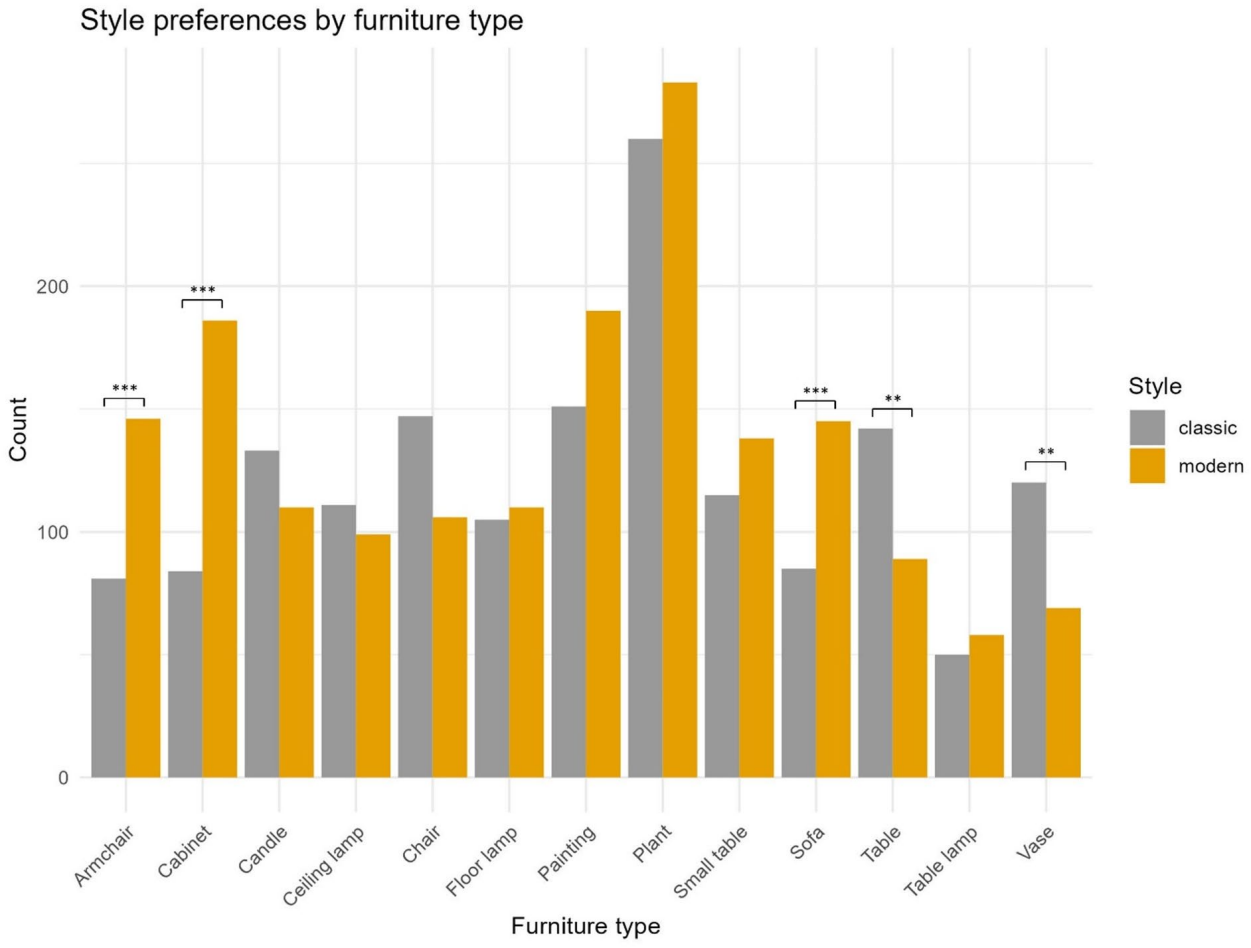
Style preferences by furniture category. Bars indicate the overall count of selected items across all participants for style (modern and classic) and category. Significant differences are based on adjusted p-values and marked with asterisks (*** for *p* < 0.001, ** for *p* < 0.01, and * for *p* < 0.05).

We repeated the chi-square test for the style of the selected items. We excluded the categories basket, cushion, and carpet from this analysis, as they did not offer options for style. The complete results of the chi-square test can be found in the supplementary material. The test showed a preference for modern furniture for armchairs (*p* < 0.001), cabinets (*p* < 0.001), and sofas (*p* < 0.001). On the contrary, it indicated a preference for classic furniture for tables (*p* = 0.006) and vases (*p* = 0.003). The total number of selected modern and classic items per furniture category is visualised in Fig. 5.

### Personality traits and preference for form and style

From the participants’ responses on the BFI-2^32^, we calculated the scores for the five trait dimensions: extraversion, agreeableness, conscientiousness, negative emotionality, and open-mindedness. We ran a multiple linear regression analysis with form as the dependent variable and the scores for the BFI-2 trait dimensions as the independent variables. The overall model was not statistically significant, *F*(5, 94) = 1.55, *p* = 0.181, indicating that the personality trait scores did not explain a significant proportion of the variance in form preferences (*R²* = 0.076, adjusted *R²* = 0.027). The regression coefficients and associated statistics for each predictor are presented in Table 1. None of the personality traits were significant, though there was a tendency for negative emotionality (*p* = 0.078) and open-mindedness (*p* = 0.070) as positive predictors of preference for selecting curved items.

We repeated this analysis for style with the percentage of selected modern items as the dependent variable. The overall model was not statistically significant, *F*(5, 94) = 0.64, *p* = 0.669, indicating that personality traits did not explain a significant proportion of the variance in style preferences (*R²* = 0.033, adjusted *R²* = −0.018). None of the predictor variables had statistically significant effects on the percentage of selected modern items. Full model statistics are provided in Table 1.

Further, we split the data into a subset containing female and male participants and repeated the multiple linear regression on each subset. We also ran the analysis on the full sample using the incomplete BFI-2 data, as the ten missing items were distributed evenly across all five trait dimensions. Results and full model statistics can be found in the supplementary material.

**Table 1:** Results of multiple linear regression models predicting form and style based on personality traits. For each predictor, the table includes the estimated regression coefficient (b), standard error (SE), t-value, and p-value.

Predictors	Estimate (b)	SE	t-value	*p*-value
**Form**				
Extraversion	−0.194	0.291	−0.666	0.507
Agreeableness	0.001	0.003	0.492	0.624
Conscientiousness	0.003	0.003	0.850	0.397
Negative emotionality	0.005	0.003	1.784	0.078
Open-mindedness	0.005	0.002	1.836	0.070
**Style**				
Extraversion	< 0.001	0.003	−0.070	0.944
Agreeableness	0.001	0.004	0.202	0.840
Conscientiousness	0.001	0.003	0.158	0.875
Negative emotionality	−0.002	0.003	−0.811	0.420
Open-mindedness	0.003	0.003	1.019	0.311

## Discussion

In this study, we introduced a novel approach to investigating design preferences using an interactive 3d web application. Participants were instructed to furnish a virtual living room according to their personal preferences while selecting from a controlled set of furniture items designed to systematically examine form and style preferences. A total of 196 participants completed the study and met the inclusion criteria.

Our analysis of general form preferences revealed a significant difference, indicating a preference for selecting angular furniture items. However, this effect was of relatively small size, with an average selection rate of 53.6% for angular items. When examining sex-related differences, the effect became slightly more pronounced. Male participants selected significantly more angular items than female participants. Further subgroup analyses showed that female participants exhibited no significant form preference, whereas male participants demonstrated a clear preference for selecting angular furniture, with an average selection rate of 57.8% for angular items. These findings suggest that the overall preference for angularity in the full sample was driven by male participants.

Although previous research has reported a preference for curvilinearity among women^8,20^, instead, we observed a stronger preference for angularity among male participants, while the female subgroup showed no significant preference. These results are consistent with the findings of Tawil et al.^7^, who reported that male participants rated angular living rooms more positively and performed better on a cognitive task in these environments compared to female participants. This connection is particularly relevant given that a part of the 3d furniture models used in our study originated from their study. However, one potential limitation must be considered. In our web application, angular furniture was consistently displayed in the top row, while curved items appeared below (see Panel 3 of Fig. 1). This layout may have introduced a selection bias, as participants might have been more likely to choose the top-row items simply due to their placement. In line with Vartanian et al.^19^, our results also indicated that participants with greater knowledge and interest in art and architecture, as measured by the VAIAK, tended to select slightly more curved furniture items. This suggests that expertise may modulate subtle form preferences even when the overall trend favours angularity.

In contrast to our findings on form preferences, we did not observe any significant differences in style preference for modern or classic furniture, even when analysing sex-related differences. This result is particularly interesting given that a previous study found that images featuring modern furniture were rated higher in beauty and liking compared to classic furniture^8^. Notably, both studies used the same 3d furniture models, yet our findings do not reflect the same preference for the modern style. A key difference between the studies lies in the presentation of the stimuli. In the study of Tawil et al.^8^, participants rated images in which only modern or classic furniture was present, whereas in our study, they had full control over their selections, allowing them to mix modern and classic styles. This suggests that while participants may prefer modern over classic furniture in isolated comparisons of static images, they might favour a balanced combination of minimalistic and decorative elements when given the freedom to furnish a space themselves. Further, our results align with previous studies that also found no difference between modern and classic furniture or rooms^6,7^, underscoring the need for future research to clarify the conditions under which style preferences become evident.

Generally, another important factor to consider when interpreting these results is the mode of presentation. Previous research has shown that design evaluations can differ depending on whether participants view static images or actively explore a space in VR^8,33^. Our web application represents an intermediate form of presentation, falling between static, image-based assessments and immersive VR experiences. While participants interacted with the application in a 2d screen environment, they still had a high degree of autonomy, similar to a VR setting. They could decide where to look, how to move, and how to explore the space, rather than being limited to static perspectives. This suggests that the stereoscopic experience of VR may not be the only factor influencing design evaluations. Instead, the level of interactivity and autonomy—whether participants engage in active exploration (navigating the space freely) or passive viewing (observing static images or videos)—could also play a critical role in shaping how environments are perceived and assessed.

To gain deeper insights, we examined form and style preferences for specific furniture items. The results of the chi-square test revealed a significant preference for angularity in cabinets, candles, carpets, cushions, and paintings, while a preference for curvature was observed only for plants. These preferences likely reflect the dominant form characteristics of these items in real life. For instance, rectangular shapes are far more common for paintings and cushions, making them the more familiar and expected choice. Interestingly, we found a preference for angularity in candles, despite the fact that rounded candles are generally more prevalent. One possible explanation is that participants may not have found the design of the curved candles appealing, potentially perceiving them as unconventional. Similarly, the preference for angular cabinets could be related to practicality, as rectangular cabinets typically provide more storage space compared to curved ones.

Interestingly, no form preference emerged for seating furniture, including sofas, armchairs, and chairs. It could have been expected that curvilinear designs would often be associated with greater comfort and therefore preferred by participants. This finding suggests that participants may recognise from experience that form alone does not determine comfort—a rectangular sofa can be just as comfortable as a rounded one, and vice versa. Alternatively, comfort may not have been a deciding factor in the furnishing process, as participants could not physically experience the furniture. Instead, their choices may have been based purely on aesthetic preference, which resulted in no clear tendency to select either of the form types.

When analysing furniture-specific style preferences, we found that armchairs, cabinets, and sofas were preferred in a modern design, whereas tables and vases were favoured in a classic style. The preference for modern seating furniture suggests that participants may associate minimalistic designs with these categories. One possible explanation is that many participants likely have modern sofas, armchairs, and cabinets in their own homes, making these styles feel more familiar and appealing in the virtual setting. However, if familiarity were the driving factor, we would have expected a similar preference for modern tables, which was not the case. One explanation could be that one of the classic table designs was the only option with four traditional legs at each corner, while the modern tables had alternative base structures. Participants may have preferred the more conventional four-legged design over the contemporary alternatives. Additionally, the second classic table was the only one featuring a glass element, which might have made it particularly appealing. A full overview of all furniture items is available in the supplementary material.

A general limitation in interpreting style preferences is that we do not know to what extent participants consciously perceived the style differences. While form distinctions are relatively straightforward, style differences may be more subtle and harder to define. As a result, participants’ preferences for certain items might have been influenced more by other design features (e.g., the structure or composition of a table) rather than by style alone.

Lastly, building on previous research linking form preferences to personality traits^25^, we examined whether similar patterns emerged in our data. Our analysis revealed no significant associations between BFI-2 personality traits and form or style preferences. However, a trend was observed for negative emotionality (*p* = 0.078) and open-mindedness (*p* = 0.070) as positive predictors of curvature preference, suggesting that individuals with higher negative emotionality and open-mindedness scores may have a slight inclination toward curved items. This contrasts with the findings of Banaei et al.^25^, who reported that higher negative emotionality was associated with a decreased preference for curvature. However, given that our results did not reach statistical significance, any direct comparisons should be interpreted with caution.

In summary, this study introduced an interactive 3d web application as a novel tool for examining furniture design preferences in a flexible, yet controlled environment. Our findings suggest a general tendency for angular over curved furniture, with sex-related differences indicating that this effect is primarily driven by male participants. While previous studies linked personality traits to form preferences, our findings did not confirm a significant relationship. Taken together, these results also highlight the importance of methodological factors, such as presentation mode, in shaping design evaluations.

## Limitations

This study has several limitations. First, the placement of furniture items within the web application was not randomised, meaning that the angular option was always positioned at the top. This could have introduced a bias toward angularity, potentially explaining the preference for angular designs. However, given that we still observed sex-related differences and form-specific preferences within the furniture categories, it is likely that any such bias did not strongly influence the data.

Another limitation is the restricted selection of furniture categories. The web application did not include certain items, such as televisions or books, which some participants might consider essential for a living room. These items were excluded because it was not feasible to create realistic curved counterparts. Generally, the findings of the item-specific analysis should be interpreted with caution, as they are contingent on the provided furniture selection. With only eight items per furniture category, the range of choices was somewhat limited, and a different set of items could potentially yield different results.

Future studies could examine patterns in furniture placement and analyse preferences for materials and fabrics. Additionally, it would be interesting to investigate whether the outdoor environment influences furnishing decisions. For instance, future research could explore whether seating furniture is more likely to be arranged facing a window when the view offers a natural scene, as opposed to an urban landscape. It is also important to consider that previous research has suggested different brain mechanisms being involved in ‘wanting’ and ‘liking’ of stimuli^34^. This distinction implies that participants may express a preference towards one form when evaluating aesthetics without necessarily wanting to include such furniture when furnishing a space, like in our web application. Exploring this divergence between ‘wanting’ and ‘liking’ could offer valuable insights into how aesthetic preferences translate into actual interior design choices. Moreover, it may make a difference whether participants imagine furnishing their own living rooms or furnishing a virtual room in digital applications, such as in a computer game, which future studies could directly compare. Generally, more research is needed to investigate the effect of presentation mode, e.g., evaluation of images and videos versus 3d spaces and the underlying mechanisms that alter the differences observed between design judgments of 2d and 3d spaces^8,33^.

## Conclusions

In this study, we introduced a novel approach to investigating design preferences—specifically form and style preferences in interior design—by using an interactive 3d web application. Participants freely furnished virtual living rooms according to their personal preferences, using a controlled set of furniture items. Our findings revealed a general preference for choosing angular furniture, driven primarily by male participants, while female participants showed no clear form preference. Additionally, we found no significant association between personality traits and form or style preferences. Overall, this study demonstrates the value of an experimental setup that grants participants a higher degree of autonomy in expressing their preferences. The proposed method offers flexibility and can be readily adapted to address related research questions in the fields of architecture and environmental psychology. Finally, these insights may inform future design practices and user-centred approaches in interior architecture, especially when considering gender-specific design tendencies.

## Supplementary Information

Below is the link to the electronic supplementary material.

Supplementary Material 1

## Data Availability

The presented dataset and screenshots from the rooms, as well as a video demonstrating the web application, can be found here: https://doi.org/10.17605/OSF.IO/27TXP.
