# Supplementary material for: Interactive room design as a tool for understanding form and style preferences
Source: Sci Rep. 2025 Oct 13;15:35578. doi: 10.1038/s41598-025-23543-5 (PMC12518833; doi:10.1038/s41598-025-23543-5)

Supplementary Material

**Age-Related Differences in Form and Style Preferences**

To test for age-related differences in form and style preferences, we correlated participants’ age with the percentage of selected curved and modern furniture. There was no significant correlation between age and percentage of selected curved furniture: r(194) = -0.001, p = 0.931. Further, there was no significant correlation between age and percentage of selected modern furniture: r(194) = -0.030, p = 0.674.

We split participants into the following age groups:

- Age 18 to 24
- Age 25 to 34
- Age 35 to 44
- Age 45 to 54
- Age 55 to 64

A Welch’s one-way ANOVA was conducted to test for differences in form preferences between the age groups. The results indicated no statistically significant difference between the five age groups, F(4, 16.56) = 0.16, p = 0.953. We repeated the test to assess differences in style preferences between age groups. The test was not significant, F(4, 17.15) = 2.56, p = 0.076, indicating that style preferences did not differ between the age groups.

**Form and Style Preferences per Furniture Category**

Tables 1 and 2 show results for the chi-square tests that were conducted to assess preferences for forms (Table 1) and style (Table 2) within the furniture categories.

| **Furniture type** | **Chi-square p-value** | **Adjusted p-value** |
| --- | --- | --- |
| Armchair | 0.014 | 0.225 |
| Basket | 0.347 | 1.000 |
| Cabinet | **< 0.001 ***** | **< 0.001 ***** |
| Candle | **0.002 **** | **0.027 *** |
| Carpet | **< 0.001 ***** | **< 0.001 ***** |
| Ceiling lamp | 0.038 | 0.615 |
| Chair | 0.014 | 0.227 |
| Floor lamp | 0.195 | 1.000 |
| Painting | **< 0.001 ***** | **< 0.001 ***** |
| Cushion | **< 0.001 ***** | **< 0.001 ***** |
| Plant | **< 0.001 ***** | **< 0.001 ***** |
| Small table | 0.285 | 1.000 |
| Sofa | 0.598 | 1.000 |
| Table | 0.056 | 0.902 |
| Table lamp | 1.000 | 1.000 |
| Vase | 0.007 | 0.114 |

*Table 1. Results for the chi-square test for preferences in form by furniture type, along with Bonferroni-adjusted p-values.*

| **Furniture type** | **Chi-square p-value** | **Adjusted p-value** |
| --- | --- | --- |
| Armchair | **< 0.001 ***** | **< 0.001 ***** |
| Cabinet | **< 0.001 ***** | **< 0.001 ***** |
| Candle | 0.140 | 1.000 |
| Ceiling lamp | 0.408 | 1.000 |
| Chair | 0.010 | 0.129 |
| Floor lamp | 0.733 | 1.000 |
| Painting | 0.023 | 1.000 |
| Small table | 0.148 | 1.000 |
| Sofa | **< 0.001 ***** | **< 0.001 ***** |
| Table | **< 0.001 ***** | **0.006 **** |
| Table lamp | 0.441 | 1.000 |
| Vase | **< 0.001 ***** | **0.003 **** |

Table 2. Results for the chi-square test for preferences in style by furniture type, along with Bonferroni-adjusted p-values.

**Personality Traits and Preference for Form and Style**

As we experienced data loss due to a technical error, we reported the analyses of personality and form and style preferences in the manuscript on a subset of N = 100 participants, where we had complete BFI data. However, the ten missing items were distributed equally amongst the traits, with two items belonging to each trait. Therefore, we also ran the analysis on all participants, however, scores for the personality traits were calculated with 10 instead of 12 items. The results of the multiple linear regression are reported in Table 3. The models could not explain a substantial amount of variance for form (R² = 0.07, adjusted R² = 0.04) and style choices (R² = 0.02, adjusted R² = - 0.001).

| **Predictors** | **Estimate (b)** | **SE** | **t-value** | **p-value** |
| --- | --- | --- | --- | --- |
| **Form** | | | | |
| Extraversion | -0.010 | 0.002 | -0.031 | 0.975 |
| Agreeableness | 0.004 | 0.003 | 1.481 | 0.140 |
| Conscientiousness | 0.002 | 0.002 | 1.050 | 0.295 |
| Negative emotionality | 0.006 | 0.002 | 2.331 | **0.021 *** |
| Open-mindedness | 0.005 | 0.002 | 2.197 | **0.029 *** |
| **Style** | | | | |
| Extraversion | -0.001 | 0.002 | -0.602 | 0.548 |
| Agreeableness | 0.001 | 0.003 | 0.221 | 0.825 |
| Conscientiousness | < 0.001 | 0.002 | 0.130 | 0.897 |
| Negative emotionality | -0.003 | 0.003 | -1.258 | 0.210 |
| Open-mindedness | 0.003 | 0.002 | 1.235 | 0.218 |

Table 3. Results of multiple linear regression models predicting form and style based on personality traits from N = 196 participants. For each predictor, the table includes the estimated regression coefficient (b), standard error (SE), t-value, and p-value. Significant differences are marked with asterisks (*** for p < 0.001, ** for p < 0.01, and * for p < 0.05).

**Personality Traits and Preference for Form and Style – Sex-related Differences**

We repeated the analysis to examine whether personality traits could predict form and style preferences on the subsets of male and female participants. Here, we again took the complete data (where we had responses on all 60 BFI-2 items), leaving us with relatively small sample sizes of 48 male and 52 female participants. For the subsample of male participants, the models could explain a variance of R² = 0.13 (adjusted R² = 0.02) for form and R² = 0.15 (adjusted R² = 0.05) for style. Complete model statistics are provided in Table 4. For the subsample of female participants, the model could explain a variance of R² = 0.05 (adjusted R² = - 0.05) for form and R² = 0.08 (adjusted R² = - 0.03) for style. Full model statistics can be found in Table 5.

| **Male Participants** | | | | |
| --- | --- | --- | --- | --- |
| **Predictors** | **Estimate (b)** | **SE** | **t-value** | **p-value** |
| **Form** | | | | |
| Extraversion | -0.001 | 0.004 | -0.236 | 0.815 |
| Agreeableness | 0.002 | 0.006 | 0.331 | 0.742 |
| Conscientiousness | < 0.001 | 0.005 | 0.022 | 0.983 |
| Negative emotionality | 0.001 | 0.005 | 0.214 | 0.832 |
| Open-mindedness | 0.012 | 0.005 | 2.274 | **0.028 *** |
| **Style** | | | | |
| Extraversion | 0.001 | 0.005 | 0.155 | 0.878 |
| Agreeableness | -0.005 | 0.006 | -0.804 | 0.426 |
| Conscientiousness | -0.007 | 0.005 | -1.334 | 0.189 |
| Negative emotionality | -0.007 | 0.005 | -1.500 | 0.141 |
| Open-mindedness | 0.011 | 0.005 | 2.106 | **0.041 *** |

Table 4. Results of multiple linear regression models predicting form and style based on personality traits for a subset of male participants (N = 48). For each predictor, the table includes the estimated regression coefficient (b), standard error (SE), t-value, and p-value. Significant differences are marked with asterisks (*** for p < 0.001, ** for p < 0.01, and * for p < 0.05).

| **Female Participants** | | | | |
| --- | --- | --- | --- | --- |
| **Predictors** | **Estimate (b)** | **SE** | **t-value** | **p-value** |
| **Form** | | | | |
| Extraversion | 0.002 | 0.003 | 0.551 | 0.585 |
| Agreeableness | -0.001 | 0.005 | -0.144 | 0.886 |
| Conscientiousness | 0.004 | 0.004 | 0.890 | 0.378 |
| Negative emotionality | 0.006 | 0.004 | 1.424 | 0.161 |
| Open-mindedness | 0.002 | 0.004 | 0.604 | 0.549 |
| **Style** | | | | |
| Extraversion | -0.002 | 0.004 | -0.611 | 0.544 |
| Agreeableness | 0.004 | 0.006 | 0.744 | 0.461 |
| Conscientiousness | 0.005 | 0.004 | 1.058 | 0.296 |
| Negative emotionality | -0.002 | 0.004 | -0.531 | 0.598 |
| Open-mindedness | -0.002 | 0.004 | -0.436 | 0.665 |

Table 5. Results of multiple linear regression models predicting form and style based on personality traits for a subset of female participants (N = 52). For each predictor, the table includes the estimated regression coefficient (b), standard error (SE), t-value, and p-value. Significant differences are marked with asterisks (*** for p < 0.001, ** for p < 0.01, and * for p < 0.05).

**Furniture Items and Categories**


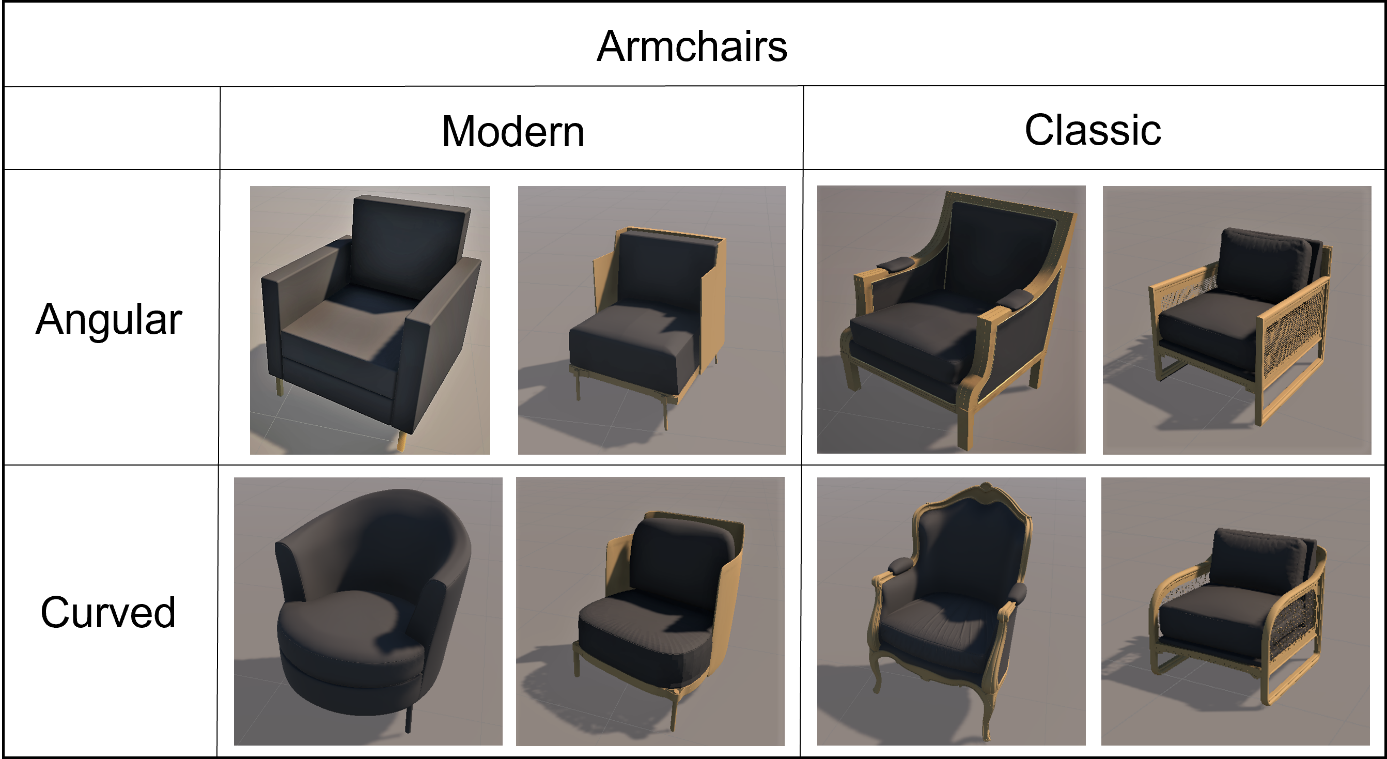


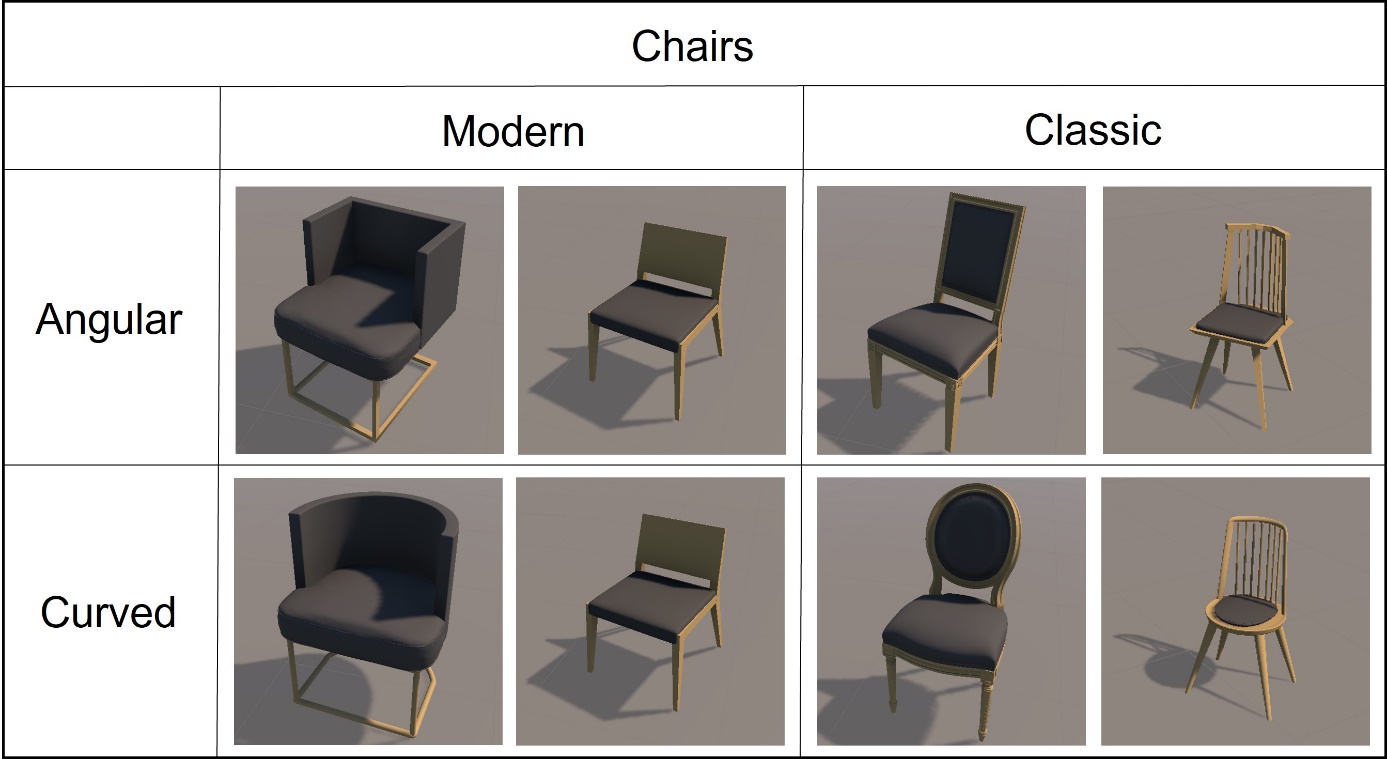


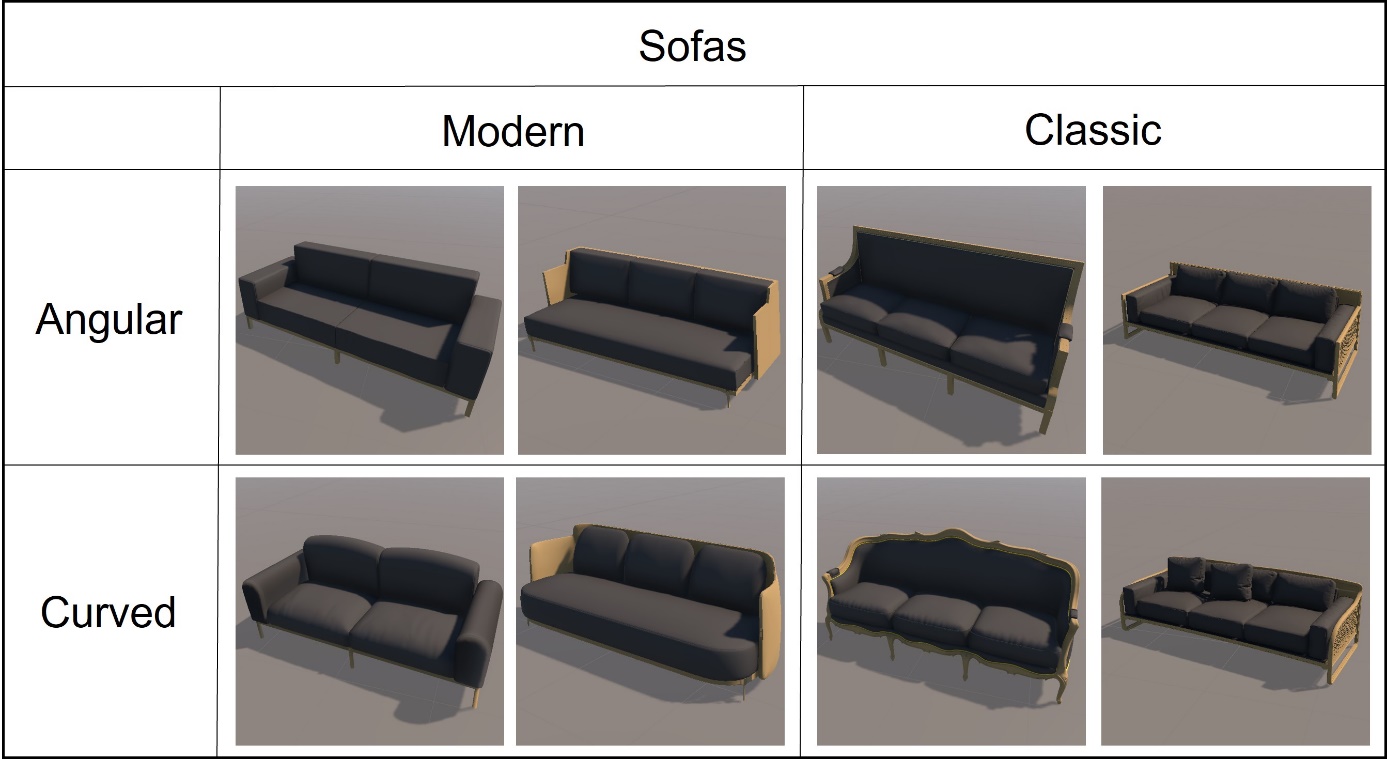


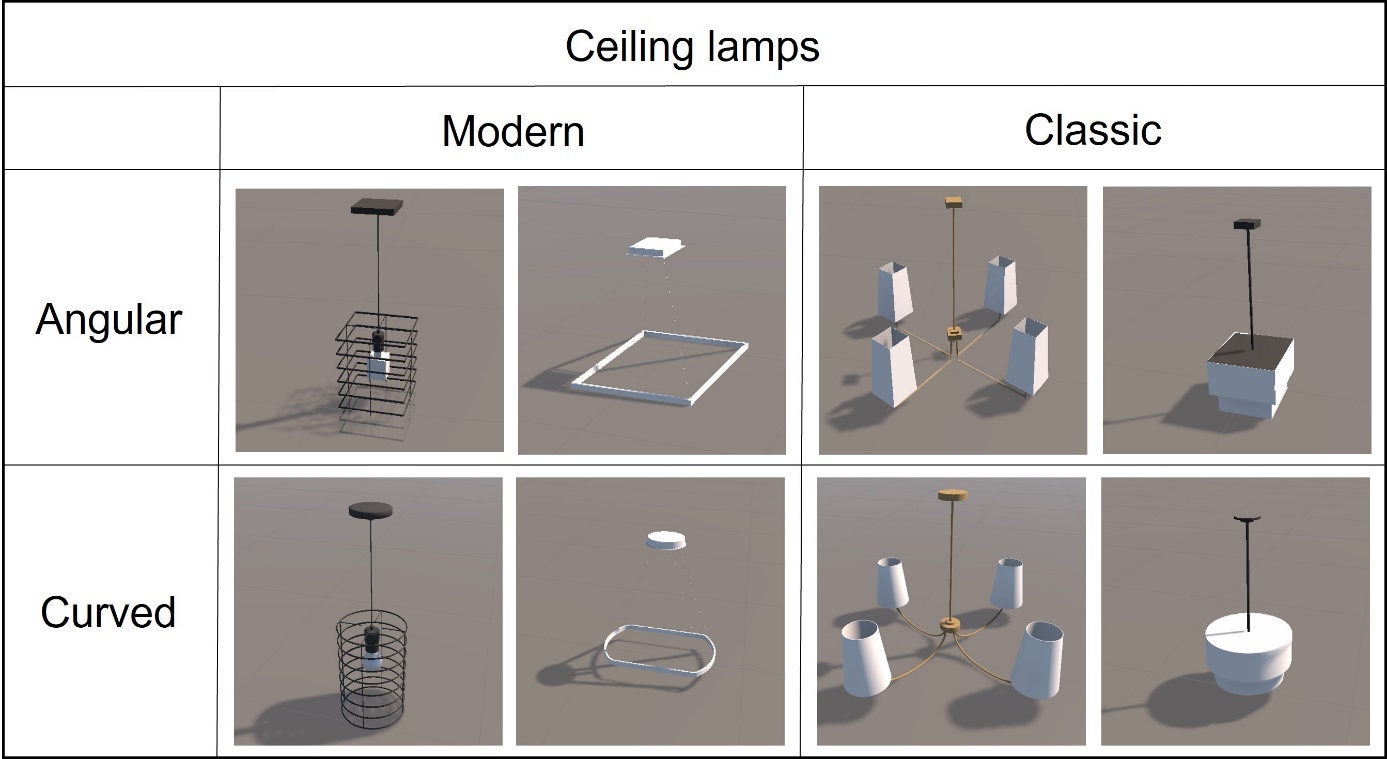


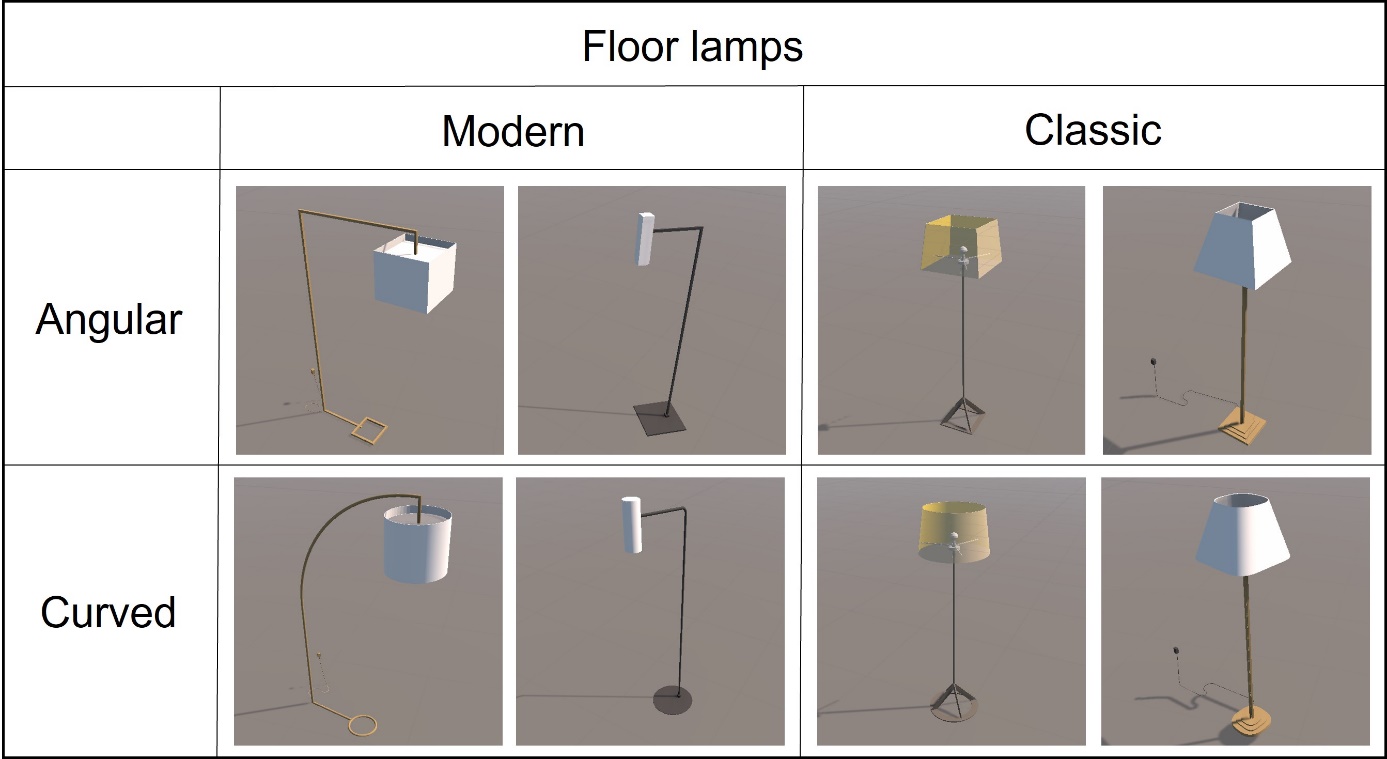


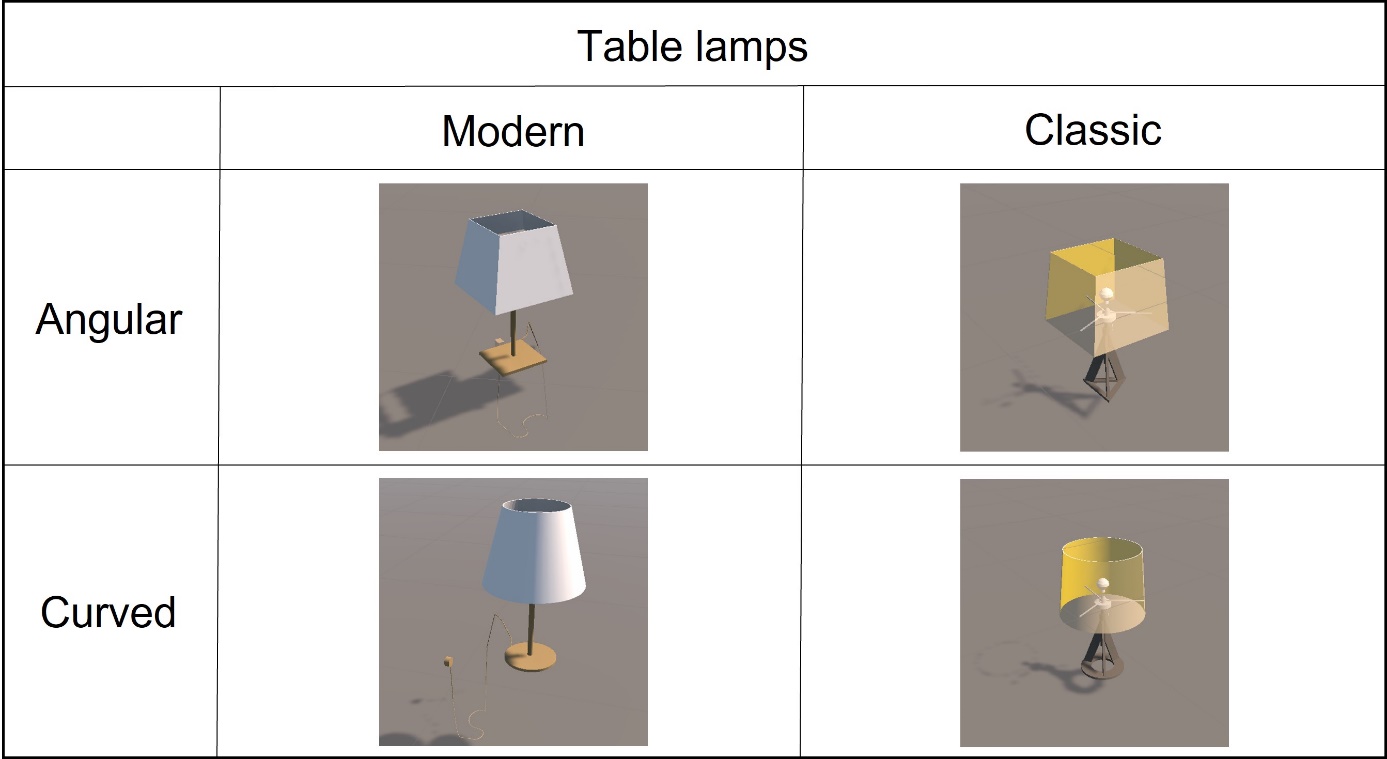


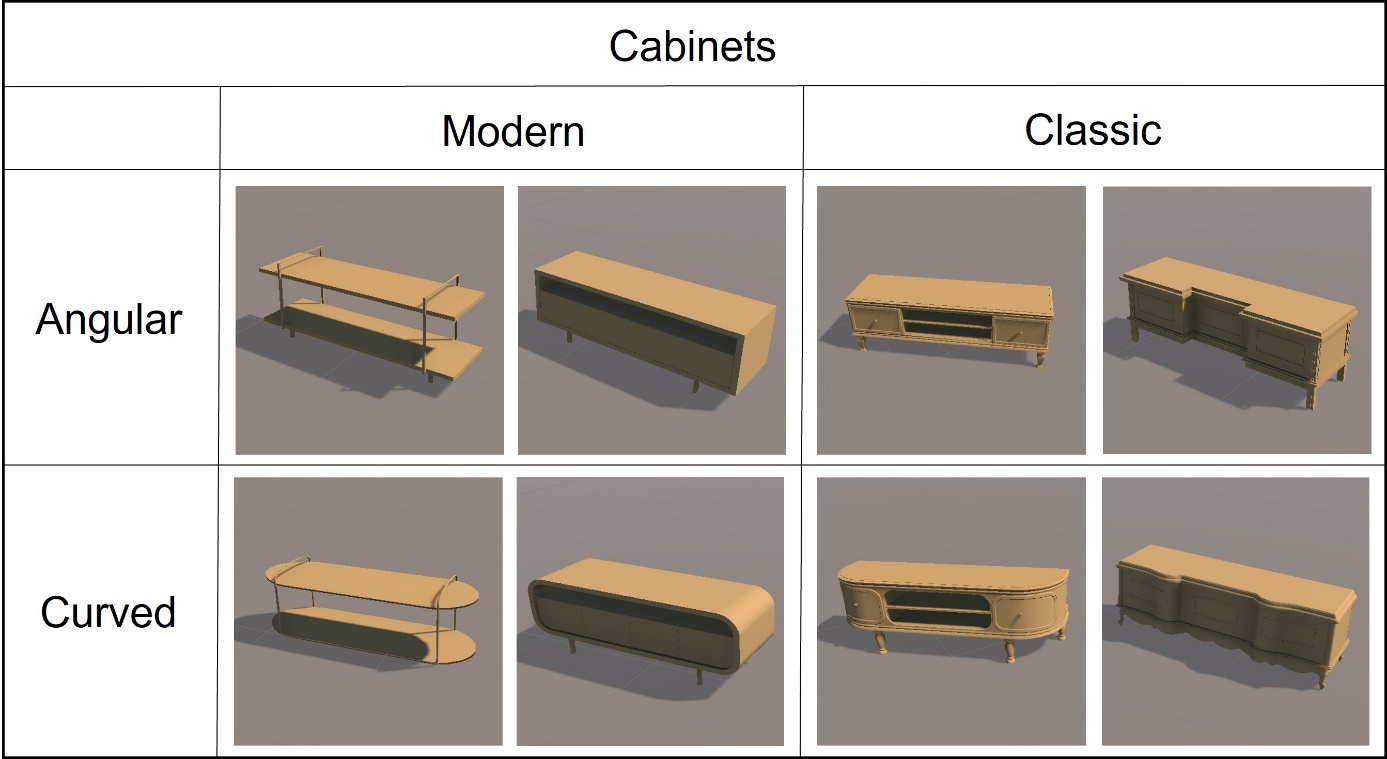


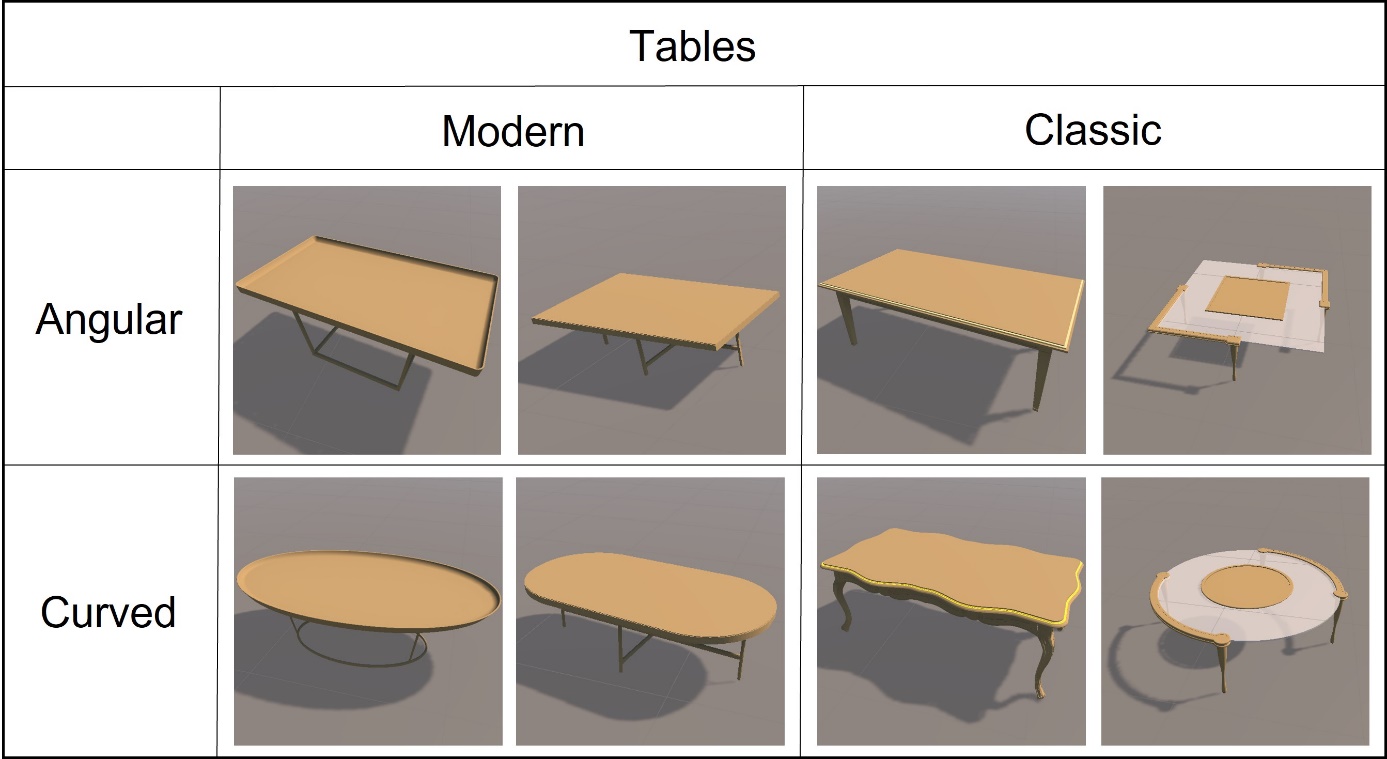


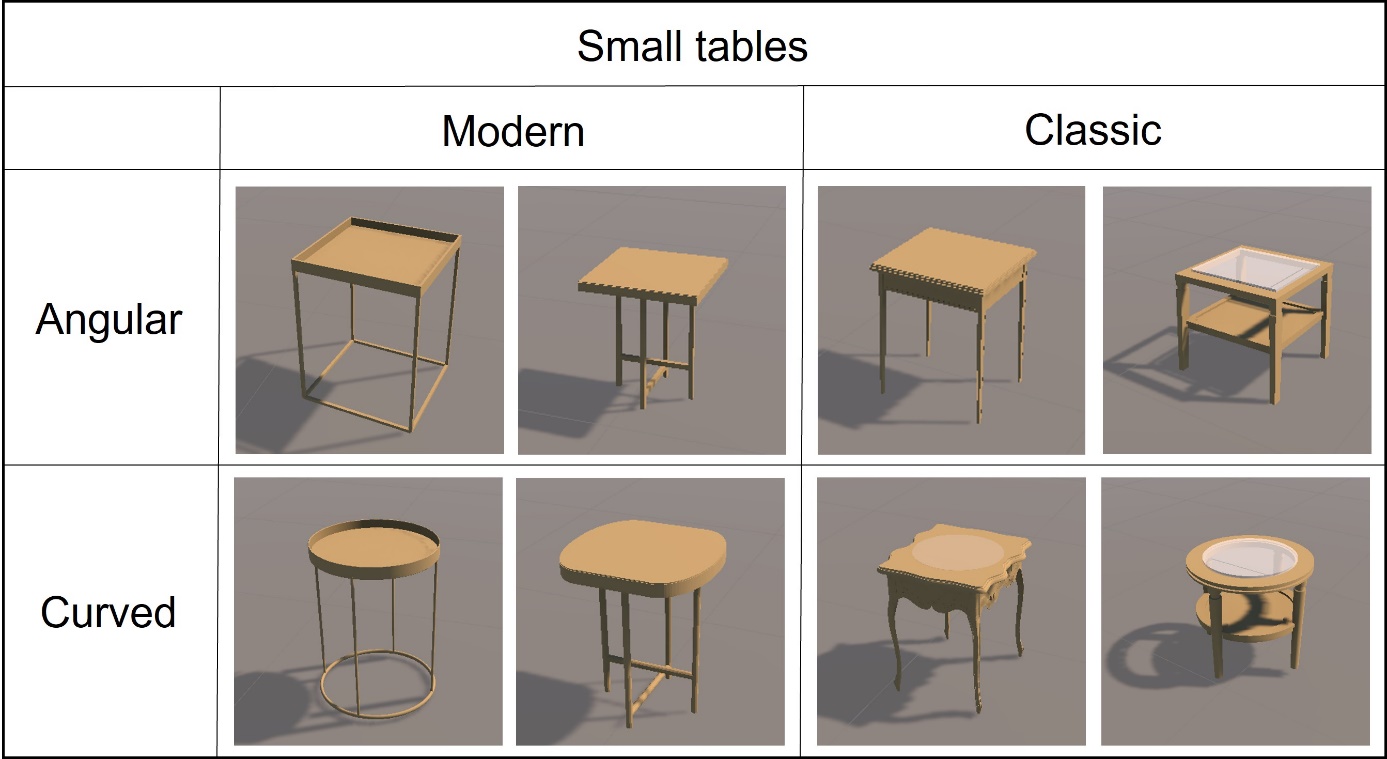


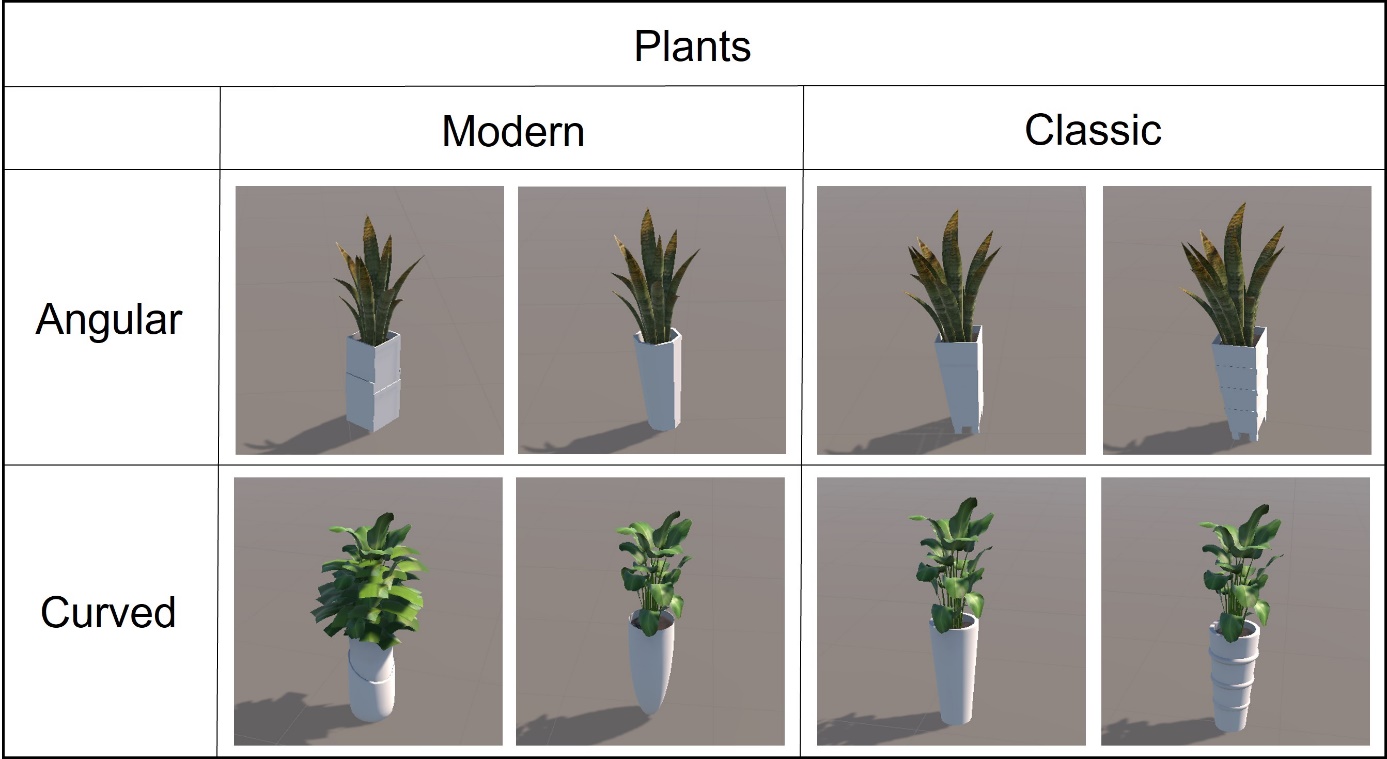


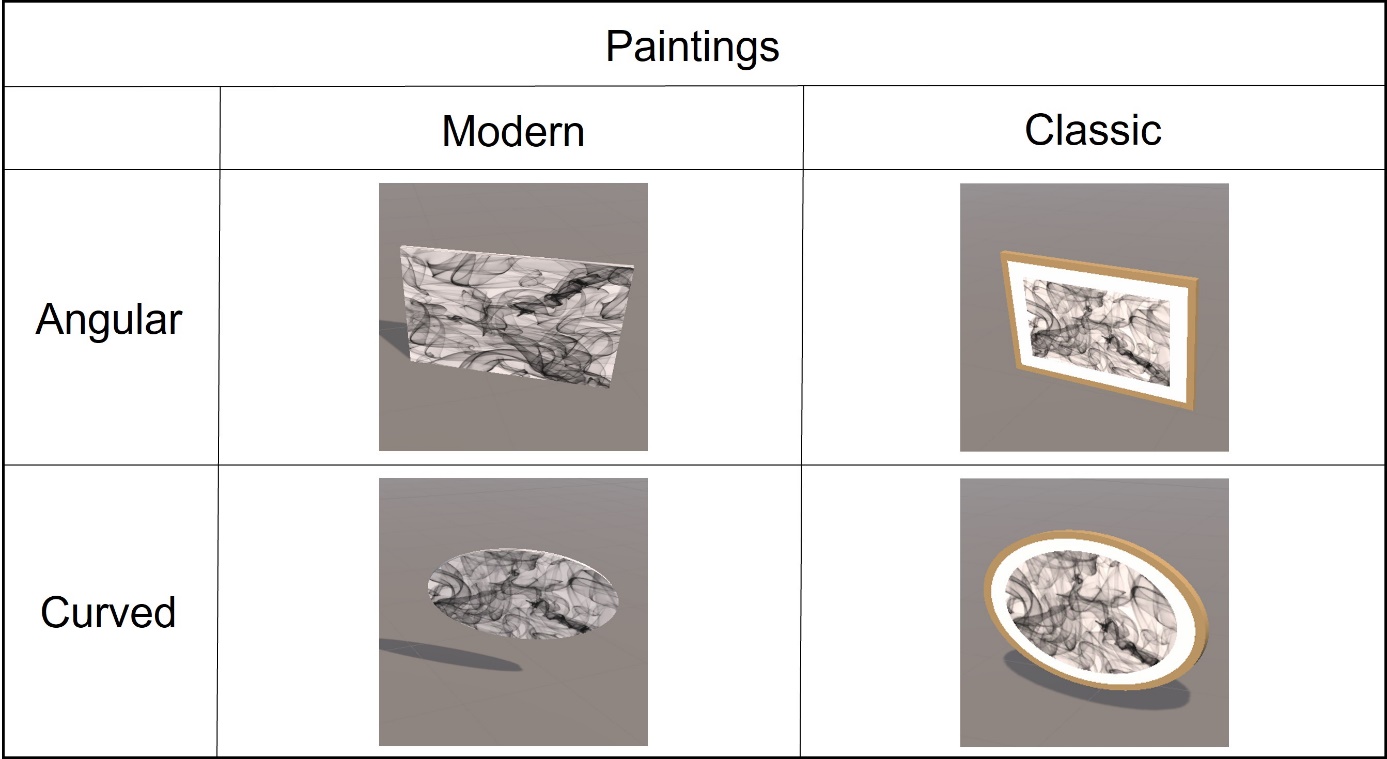


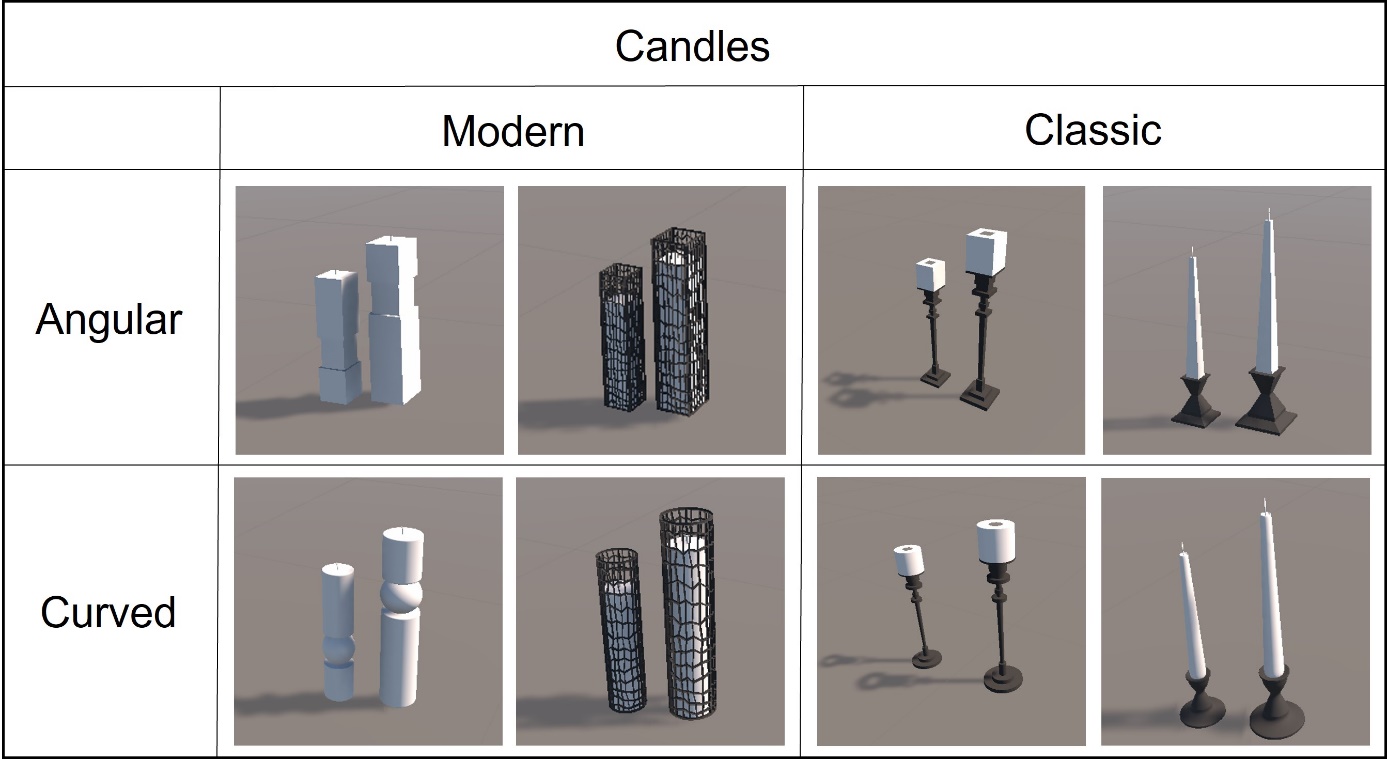


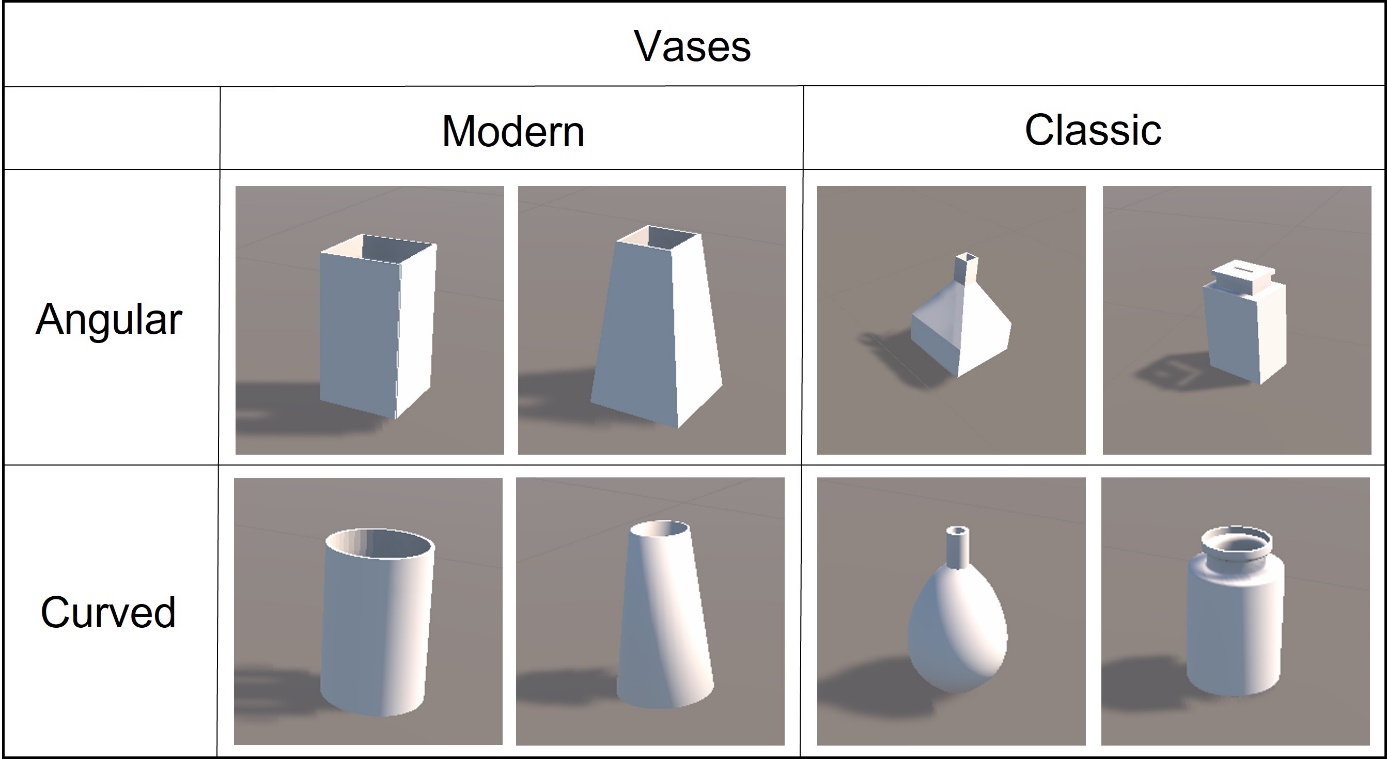


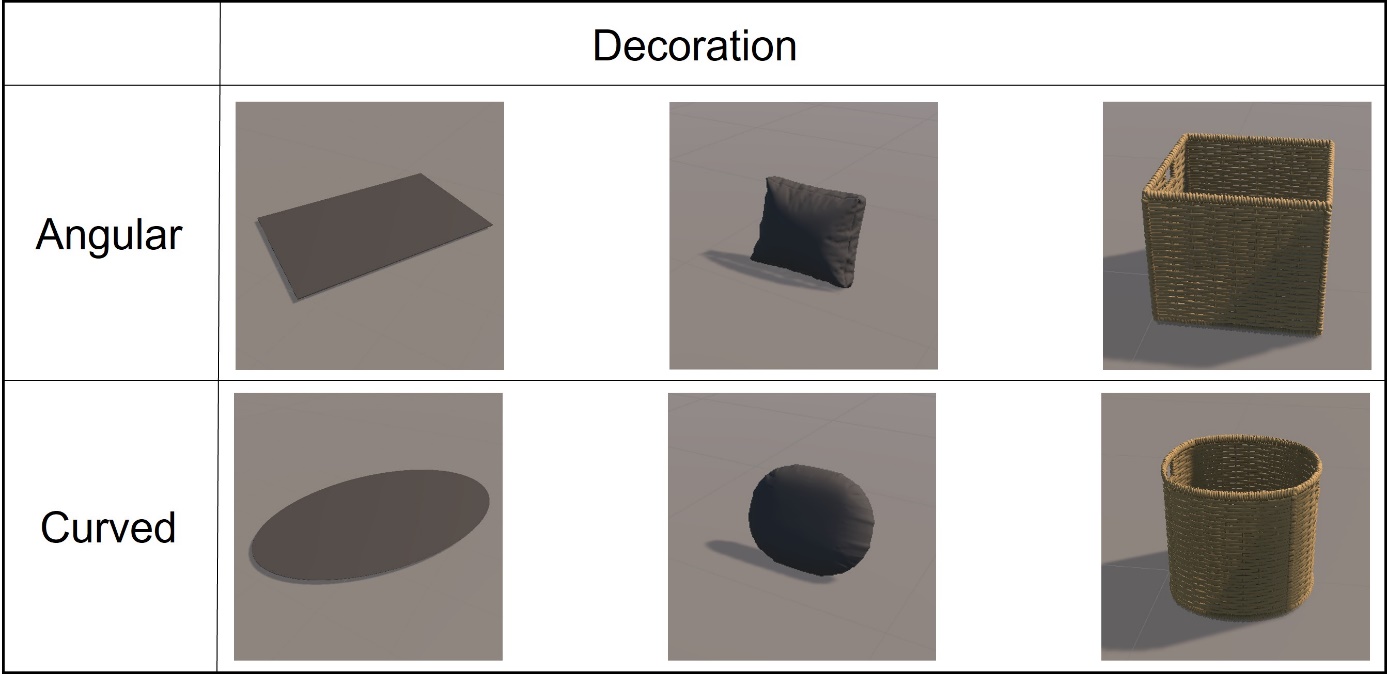

Supplement: Supplementary file 1 — Supplementary Material 1 [file 41598_2025_23543_MOESM1_ESM.docx]
